# Application of CIBERSORTx and BayesPrism to deconvolution of bulk RNA-seq data from human myocardium and skeletal muscle

**DOI:** 10.1016/j.heliyon.2025.e42499

**Published:** 2025-02-10

**Authors:** Marcella Conning-Rowland, Chew W. Cheng, Oliver Brown, Marilena Giannoudi, Eylem Levelt, Lee D. Roberts, Kathryn J. Griffin, Richard M. Cubbon

**Affiliations:** Leeds Institute of Cardiovascular and Metabolic Medicine, The University of Leeds, Leeds, United Kingdom

**Keywords:** RNA-Seq, Deconvolution, Myocardium, Skeletal muscle, Age, Sex

## Abstract

RNA-sequencing (RNA-seq) is an important tool to explore molecular mechanisms of disease. Technological advances mean this can be performed at the single-cell level, but the large sample sizes needed in clinical studies are currently prohibitively expensive and complex. Deconvolution of bulk RNA-seq offers an opportunity to bridge this gap by defining the cell lineage composition of samples. This approach is widely used in immunology studies, but currently there are no validated pipelines for researchers analysing human myocardium or skeletal muscle. Here, we describe the application and *in silico* validation of two pipelines to deconvolute human right atrium, left ventricle and skeletal muscle bulk RNA-seq data. Specifically, we have defined the major cell lineages of these tissues using single cell/nucleus RNA-seq data from the Heart Cell Atlas, which are then applied during deconvolution using the CIBERSORTx or BayesPrism deconvolution packages. Both pipelines gave robust estimates of the proportion of all major cell lineages in these tissues. We demonstrate their value in defining age- and sex-differences in tissue composition using bulk RNA-seq data from the GTEx consortium. Our validated pipelines can be rapidly applied by researchers working with existing or novel bulk RNA-seq of myocardium or skeletal muscle to gain novel insights.

## Highlights

• Deconvolution of bulk RNA-seq can inform about the cell lineage content of samples.
• We apply two deconvolution methods to human myocardium and skeletal muscle.
• *In silico* validation shows robust estimates of all major cell lineage abundances.
• We use this to demonstrate age- and sex-differences in the GTEx cohort.
• All content needed to apply the pipelines is provided for other researchers to use.

## 1 Introduction

Single-cell RNA-seq (scRNA-seq) has recently become an important tool to discern heterogeneous patterns of differential gene expression between and within the many cell lineages of which tissues are composed [1]. This has led to new insights regarding fundamental biological processes and is increasingly informing researchers about the implications of disease. However, the cost and complexities of sample preparation and data processing can be prohibitive, especially in the context of clinical studies with substantial between-donor variability. Whilst tissue dissociation methods can be used to isolate cell lineages of interest for bulk RNA-seq, these approaches may introduce dissociation artefacts, are resource intensive and require suitable cell surface marker expression to specifically capture cells of interest. Moreover, cells exceeding 30 μm in diameter (e.g. adult cardiomyocytes) are challenging to dissociate and process with high-throughput scRNA-seq methods. Whilst single nuclear RNA-seq (snRNA-seq) can circumvent these challenges, it remains expensive and technically challenging, along with neglecting cytoplasmic and mitochondrial resident mRNA [2]. Deconvolution of whole tissue bulk RNA-seq data offers an alternative approach to derive some of these insights, ranging from cell lineage composition within the sample to inferring gene expression within specific cell lineages [3]. Beyond avoiding the technical and resource limitations of other approaches, deconvolution also allows use of existing bulk RNA-seq datasets, adding value to the large volume of publicly available data.

Many tools are now available for bulk RNA-seq deconvolution, such as TIMER, MuSiC and CIBERSORTx [[4], [5], [6]]. Whilst each has pros and cons, CIBERSORTx has been demonstrated to robustly define cell lineage proportions in a range of bulk RNA-seq deconvolution scenarios [7], alongside a well-constructed user interface that makes data processing relatively simple [8,9]. Like many deconvolution approaches, it relies on the creation of ‘signature matrices’, often from scRNA-seq data, to define the expected gene expression patterns of major cell lineages within the tissue. Complex algorithms are then used to apply a relevant signature matrix to bulk RNA-seq datasets to infer their cell lineage composition, and even to predict gene expression profiles within these lineages. Most applications of this approach have so far focussed on tumours and the immune system. For example, the validated LM22 signature matrix generated during the development of CIBERSORT, the precursor to CIBERSORTx, has been used to profile circulating and tumour infiltrating immune cells [8,10]. Notably, inferring the immune cell composition of tumour samples using deconvolution of bulk RNA-seq data has allowed the identification of patients at risk of adverse outcomes [11]. More recently, the BayesPrism deconvolution package has been released and demonstrated by its creators to outperform CIBERSORTx, and other approaches, in predicting cell lineage proportions of tissues with known composition [12]. This improved performance may reflect its use of Bayesian inference to account for technical sources of variation in datasets; notably this is much more computationally efficient than CIBERSORTx and so can be applied using an R package without reliance on a central server.

Many tissues exhibit important differences in cell lineage composition related to physiological and pathological factors. For example, the cellular composition of the mouse heart varies between sexes and in the context of diabetes mellitus has increased abundance of resident mesenchymal cells and cardiac fibroblasts [13,14]. Evaluating such changes in tissue composition using bulk RNA-seq data can add valuable contextual information to studies. For example, significant changes in gene expression may occur as a result of expansion or loss of a cell lineage which highly expresses gene(s) found amongst differentially expressed hits. Moreover, differences in composition may reflect important differences in tissue structure and function that can be prioritised for future investigation using complementary approaches. Whilst deconvolution cannot offer the level of insight provided by scRNA-seq, it allows us to learn more from existing and newly generated bulk RNA-seq datasets, often in sample sizes that we cannot expect to see from scRNA-seq experiments in the near future. However, the lack of validated pipelines limits the rapid adoption of this approach. Indeed, there are no published examples of this approach in the analysis of myocardium or skeletal muscle. Therefore, we set out to produce validated CIBERSORTx and BayesPrism pipelines for the research community to gain wider insights from analyses of human myocardium and skeletal muscle.

## 2 Methods

### 2.1 CIBERSORTx signature matrix construction

To generate signature matrices, we used sc/snRNA-seq raw count data from the Heart Cell Atlas version 1 (https://www.heartcellatlas.org/v1.html), downloaded via the UCSC browser (https://cells.ucsc.edu/?ds=heart-cell-atlas). This project set out to profile all major cell types in multiple anatomical regions of the human heart [15]. It included skeletal muscle samples from the intercostal muscle of 5 healthy individuals (2 female, 3 male) aged 60–75 and myocardial samples from 14 healthy individuals (7 male, 7 female) aged 40–75 [15]. We used cells from the “Apex”, “Right Atrial” and “Skeletal Muscle” regions to generate signature matrices for left ventricle (LV), right atrium (RA) and skeletal muscle, respectively. We focussed on these cardiac chambers as most publicly available myocardial bulk RNA-seq data arise from these sites.

To generate the single cell reference file, first we removed cells classified by the Heart Cell Atlas investigators as lacking an identity or representing doublets; duplicate gene names were also removed. We included all cell lineages identified and annotated by the Heart Cell Atlas investigators, with the exception of the myocardial mesothelial cell population, which was not included as there were insufficient cells in the dataset to accurately represent and validate them. From all remaining cell lineages curated by the Heart Cell Atlas in our tissues of interest, 200 cells were randomly selected using the sample function in R (Version 4.2.0) to represent their lineage. The CIBERSORTx authors have shown that even 20 cells per lineage provides robust deconvolution performance and advise avoiding much larger values to minimise computational requirements [6,11]. The output generated a.tsv file for each tissue, with gene names in the first column and all subsequent columns giving raw counts data for each of the single cells selected to represent each lineage, with column headers denoting the lineage and cell number (Supplemental Files 1-3). These single cell reference files were uploaded to CIBERSORTx (https://cibersortx.stanford.edu/) [11]. The analysis module used was “1. Create Signature Matrix”. Analysis Mode used was “Custom”, Input Data type “scRNA-seq”. Quantile normalisation was disabled, as recommended by the CIBERSORTx authors for RNA-seq data. The remaining options used were the default settings: minimum expression 0.75; sampling 0.5; kappa 999; q-value 0.01; minimum number of barcode genes 300 minimum; maximum number of barcode genes 500. The CIBERSORTx authors have shown that including >500 genes to represent each lineage does not lead to improved deconvolution performance [6,11]. Filtering of non-hematopoietic genes from signature matrix was disabled. This process generated signature matrices for each tissue of interest (.txt files), alongside source GEP files (.txt files) to be used for batch correction (Supplemental Files 4-9). All contained the cell lineages: fibroblasts, endothelial cells, lymphoid cells, myeloid cells, neuronal cells, pericytes, smooth muscle cells and myocytes (defined as atrial cardiomyocytes and ventricular cardiomyocytes in the right atrial and left ventricular matrices, respectively). The cardiac signature matrices additionally included adipocytes and neuronal cells, whilst the skeletal muscle signature matrix included satellite cells.

### 2.2 *In silico* validation

#### 2.2.1 Generation of synthetic tissues

To test the performance of our signature matrices *in silico*, synthetic tissues of known composition were generated using snRNA-seq data from the Heart Cell Atlas [15]; data from cells used to generate our signature matrices were excluded to avoid bias. One hundred simulated tissues were produced following the method of Luca et al., generating highly diverse cellular compositions that allowed us to assess the performance of CIBERSORTx and our signature matrices across a very broad spectrum of input material [16]. To establish the desired range of simulated cellular composition, first we used CIBERSORTx to apply our signature matrices to publicly available RA (n = 429), LV (n = 423) and skeletal muscle (n = 803) bulk RNAseq data from the Genotype Tissue Expression project (GTEx) resource [17]. From the predicted cell lineage abundance in all samples, we calculated the GTEx population mean and standard deviation for each cell lineage in the three tissues of interest. For each tissue, we then defined the desired compositions of 100 synthetic tissues composed of 1500 cells with a distribution of cell lineage proportions reflecting the GTEx mean, but with a standard deviation at least three times that of GTEx to assess extremes of tissue composition. Again, this follows the approach of Luca et al., except that they used 1000 cells per synthetic tissue [16]. Specifically, this process applied the rnorm function of base R using a mean defined from GTEx and a standard deviation defined as the greater of either 3x that observed in GTEx or 0.25. Negative values were replaced with 0. Where the desired cell fractions required more cells than were available from the Heart Cell Atlas snRNA-seq data, this was reduced to the maximum possible number of cells available. The desired proportions of all cell lineages in each synthetic tissue was summed and then each normalised to give a sum of 1 in each tissue. These proportions were used to define the number of randomly selected cells of each lineage chosen from the Heart Cell Atlas to generate 100 synthetic tissues each composed of 1500 cells. Within these synthetic tissues the summed expression of each gene across all cells was calculated to generate the final synthetic tissue datasets.

#### 2.2.2 Synthetic tissue deconvolution with CIBERSORTx

Our synthetic RA, LV and skeletal muscle datasets were deconvoluted using CIBERSORTx, applying our signature matrices and ‘S mode’ batch correction. The predicted proportions of cell lineages in each sample was the compared against its known composition. Scatterplots were used to illustrate these data, and linear regression to quantify agreement between predicted and known composition. A step-by-step protocol for the full CIBERSORTx workflow is provided in Supplementary File 10.

#### 2.2.3 Synthetic tissue deconvolution with BayesPrism

The BayesPrism R package (v2.2.2) was applied according to the authors' instructions in the package vignette (https://github.com/Danko-Lab/BayesPrism) [12]. For the single cell matrix, ‘Cell Type’ labels were provided according to the cell lineages assigned to cells by the Heart Cell Atlas authors, as noted earlier. We used the same 200 cells per lineage that were applied in our CIBERSORTx analyses to facilitate direct comparison of model predictions. We used identical ‘Cell State’ labels to those of ‘Cell Type’ labels since identification of subsets within major lineages was not required. As recommended by the authors, genes located on the X and Y chromosomes were excluded, since the sex composition was not identical between reference (Heart Cell Atlas) and application (synthetic or GTEx) datasets. We also excluded ribosomal and mitochondrial gene groups, as recommended, to remove noise and increase computation speed. Genes expressed in fewer than 5 cells were also excluded. Deconvolution was performed using protein coding genes in the reference, to reduce batch effects and increase speed. When creating a ‘Prism object’, key type was set to null as there were no known malignant cell types. The removal of outliers was used performed according to default settings of “outlier.cut = 0.01, outlier.fraction = 0.1” where genes are filtered when their expression fraction is greater than the outlier cut setting. The posterior mean of cell type fractions predicted in our synthetic tissues was compared with their known compositions, as described above for CIBERSORTx. The R script used for our BayesPrism workflow is provided in Supplementary File 11.

### 2.3 Deconvolution of publicly available bulk RNAseq data from the GTEx project

The GTEx project has collated bulk RNA-seq data across 54 human tissues in order to study gene expression and its regulation (https://gtexportal.org/home/) [17]. We used the RA (n = 429), LV (n = 423) and skeletal muscle (n = 803) data from release version 8 to illustrate the performance of our CIBERSORTx and BayesPrism pipelines in identifying known and novel associations of age and sex with tissue cellular composition, as already described for synthetic tissues. In our primary analysis, the same 200 cells per lineage that were used in our CIBERSORTx signature matrices were applied in BayesPrism; in a sensitivity analysis, all cells from the Heart Cell Atlas (excluding myocardial mesothelial cells) were applied in BayesPrism to define if this altered its tissue composition predictions. Age and sex differences in cell lineage proportions were tested with Kruskal-Wallis and Mann-Whitney U tests, respectively. Age was categorised into groups (20–29, 30–39, 40–49, 50–59, 60–69, 70–79 years) as per the formatting of publicly available GTEx metadata. All statistical tests were adjusted for false discovery rate using the Benjamini-Hochberg approach.

## 3 Results

### 3.1 In silico validation of CIBERSORTx and BayesPrism

For RA, LV and skeletal muscle, first we compared the known and predicted proportions of all cell lineages in 100 synthetic tissues assessed using CIBERSORTx and our novel CIBERSORTx signature matrices (Supplemental Files 1-3). Linear regression revealed robust concordance of known and predicted data, with R^2^ of at least 0.9 for all cell lineages across tissues (Table 1). Scatterplots of these data also demonstrate excellent concordance (Supplemental Figs. 1–3), although with modest under- (e.g. RA adipocytes) or over-estimation (e.g. skeletal muscle myocytes) in some instances. We then went on to use BayesPrism to deconvolute the same 100 synthetic tissues; this package does not require signature matrices, but we trained it using the same 200 cells per lineage that we applied in generating CIBERSORTx signature matrices, facilitating comparison of pipelines. Again, there was strong concordance between known and predicted data, with R^2^ of at least 0.9 for all cell lineages across tissues (Table 1). Scatterplots of these data again demonstrate excellent concordance (Supplemental Figs. 4–6), although with modest under- or over-estimation in some instances. Overall, these data indicate robust deconvolution of cell lineage proportions using both CIBERSORTx and BayesPrism. Notably, this performance was maintained in synthetic tissues with compositions substantially deviating from normal physiology (and probably exceeding all but the most extreme pathological states), suggesting their performance is not sensitive to input conditions.

**Table 1:** *In silico* validation of signature matrices.

Lineage	CIBERSORTx			BayesPrism		
	Skeletal Muscle	Left Ventricle	Right Atrium	Skeletal Muscle	Left Ventricle	Right Atrium
Adipocyte	–	0.99	0.98	–	0.98	0.98
Atrial Cardiomyocyte	–	–	0.97	–	–	0.97
Ventricular Cardiomyocyte	–	0.98	–	–	0.98	–
Myocyte	0.98	–	–	0.99	–	–
Endothelial	0.99	0.96	0.96	0.99	0.97	0.96
Fibroblast	0.99	0.98	0.97	0.99	0.97	0.95
Lymphoid	0.99	0.98	0.98	0.99	0.98	0.98
Myeloid	0.99	0.98	0.97	0.99	0.98	0.96
Neuronal	–	0.9	0.97	–	0.96	0.95
Pericytes	0.99	0.92	0.97	0.99	0.96	0.96
Satellite	0.99	–	–	0.99	–	–
Vascular Smooth Muscle	0.99	0.97	0.96	0.99	0.96	0.96

Legend: Correlation (presented as R^2^) between known and predicted cell lineage proportions in 100 synthetic tissues using CIBERSORTx and BayesPrism pipelines.

### 3.2 Deconvolution of bulk RNA-seq to define age and sex associations with tissue composition

**Fig. 1:**
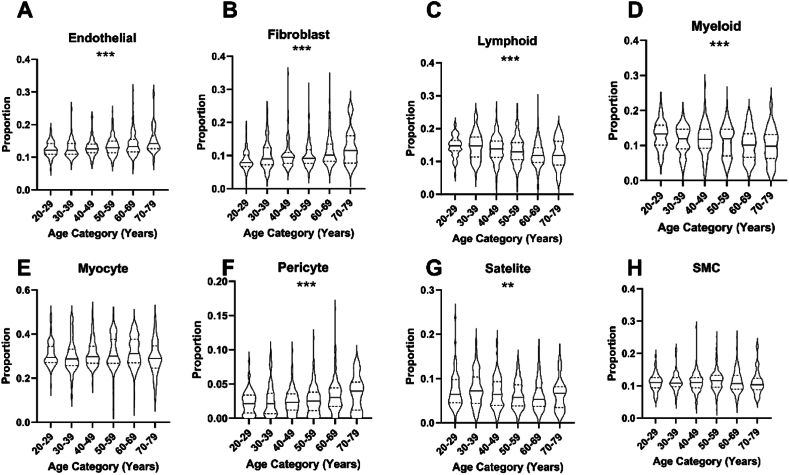
Age-associated differences in skeletal muscle cell lineage composition in the GTEx cohort using CIBERSORTx. Deconvoluted skeletal muscle cell lineage proportions according to age category in GTEx data defined using our skeletal muscle signature matrix and CIBERSORTx. SMC = Smooth muscle cells. ∗ indicate level of statistical significance of difference between groups as determined by Kruskal Wallis. ∗∗ indicates FDR ≤0.01, ∗∗∗ indicates FDR ≤0.001.

**Fig. 2:**
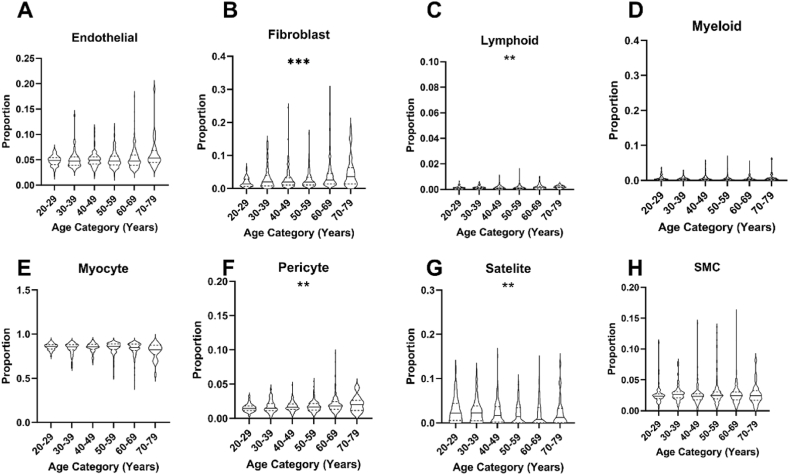
Age-associated differences in skeletal muscle cell lineage composition in the GTEx cohort using BayesPrism. Deconvoluted skeletal muscle cell lineage proportions according to age category in GTEx data defined using our BayesPrism pipeline. SMC = Smooth muscle cells. ∗ indicate level of statistical significance of difference between groups as determined by Kruskal Wallis. ∗∗ indicates FDR ≤0.01, ∗∗∗ indicates FDR ≤0.001.

**Fig. 3:**
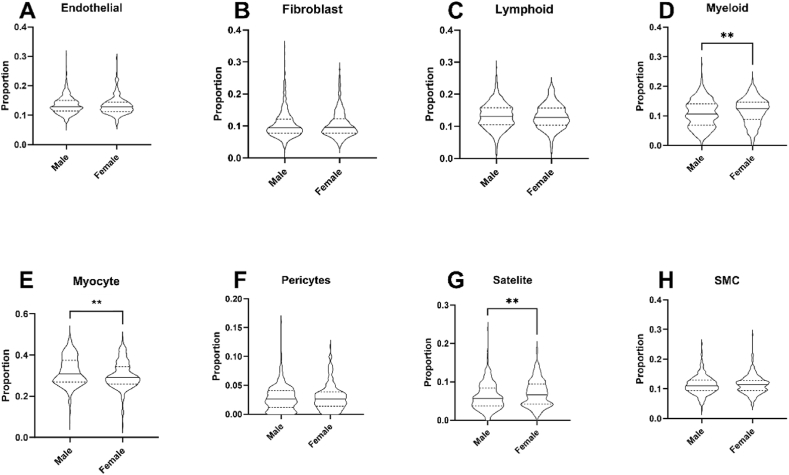
Sex-associated differences in skeletal muscle cell lineage composition in the GTEx cohort using CIBERSORTx. Deconvoluted skeletal muscle cell lineage proportions according to sex in GTEx data defined using our skeletal muscle signature matrix and CIBERSORTx. SMC = Smooth muscle cells. ∗ indicate level of statistical significance of difference between groups as determined by Wilcoxon test. ∗∗ indicates FDR ≤0.01, ∗∗∗ indicates FDR ≤0.001.

**Fig. 4:**
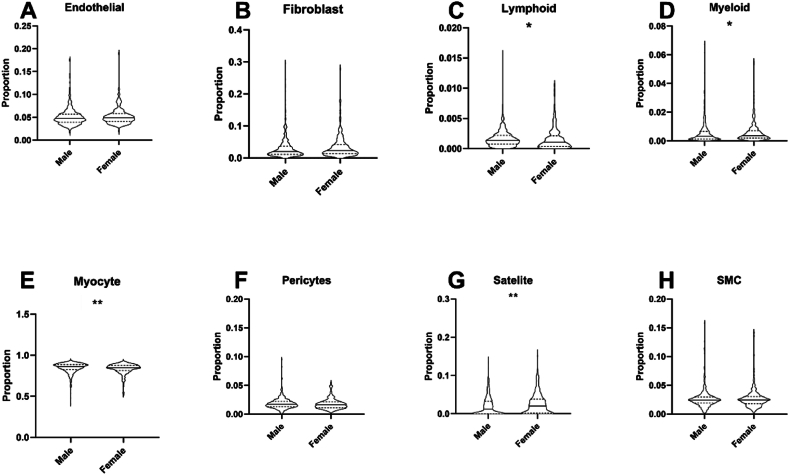
Sex-associated differences in skeletal muscle cell lineage composition in the GTEx cohort using BayesPrism. Deconvoluted skeletal muscle cell lineage proportions according to sex in GTEx data defined using our BayesPrism pipeline. SMC = Smooth muscle cells. ∗ indicate level of statistical significance of difference between groups as determined by Wilcoxon test. ∗∗ indicates FDR ≤0.01, ∗∗∗ indicates FDR ≤0.001.

We used RA, LV and skeletal muscle bulk RNA-seq data from the GTEx project to explore the performance of our CIBERSORTx and BayesPrism deconvolution pipelines in defining age and sex associations with tissue composition. Table 2 shows the age and sex profiles of the tissue donors. Analysis of skeletal muscle using CIBERSORTx revealed an increasing proportion of fibroblasts, pericytes and endothelial cells with advancing age; conversely, lymphoid, myeloid and satellite lineages decreased with advancing age (Fig. 1 and Table 3). When applying BayesPrism, directionally concordant associations were found for fibroblasts, pericytes and satellite cells (Fig. 2 and Table 4), but not for endothelial, myeloid or lymphoid cells. However, BayesPrism found a substantially higher proportion of myocytes and lower proportion of most other lineages, especially lymphoid and myeloid cells, than CIBERSORTx. When comparing sexes using CIBERSORTx, we noted a higher proportion of myeloid and satellite cells, along with a lower proportion of myocytes, in women (Fig. 3 and Table 5). BayesPrism concurred on the sex differences for myeloid cells, satellite cells and myocytes, along with suggesting a lower proportion of lymphoid cells in women (Fig. 4 and Table 5), although this lineage was rare (predicted at around 0.1 % of cells). Comparison of the lineage proportions predicted by CIBERSORTx and BayesPrism, we noted modest agreement with R^2^ values ranging from 0.21 for lymphoid cells to 0.76 for fibroblasts (Supplemental Fig. 7).

**Table 2:** Demographic characteristics of participants from GTEx study.

	Total	Skeletal Muscle	Left Ventricle	Right Atrium
		100 (786)	100 (429)	100 (425)
Sex	Female	32.3 (254)	32.2 (138)	31.8 (135)
	Male	67.7 (532)	67.8 (291)	68.2 (290)
Age	20–29	8.5 (67)	5.1 (22)	3.5 (15)
	30–39	8.1 (64)	6.1 (26)	4.2 (18)
	40–49	15.5 (122)	15.4 (66)	14.6 (62)
	50–59	31.7 (249)	35.4 (152)	35.8 (152)
	60–69	32.8 (258)	34.7 (149)	37.9 (161)
	70–79	3.3 (26)	3.3 (14)	4 (17)

Legend: Data presented as % (n).

**Table 3:** Age differences in lineage composition of myocardium and skeletal muscle in GTEx study defined using CIBERSORTx.

		20–29	30–39	40–49	50–59	60–69	70–79	FDR
**Skeletal Muscle**	Endothelial	0.122 (0.032)	0.121 (0.034)	0.126 (0.026)	0.13 (0.035)	0.134 (0.038)	0.142 (0.040)	<0.001
	Fibroblast	0.079 (0.034)	0.09 (0.051)	0.095 (0.033)	0.092 (0.042)	0.101 (0.052)	0.116 (0.083)	<0.001
	Lymphoid	0.148 (0.030)	0.147 (0.061)	0.138 (0.051)	0.13 (0052)	0.118 (0.046)	0.118 (0.071)	<0.001
	Myeloid	0.133 (0.057)	0.119 (0.057)	0.117 (0.055)	0.119 (0.076)	0.101 (0.067)	0.098 (0.068)	<0.001
	Myocyte	0.295 (0.072)	0.288 (0.075)	0.297 (0.076)	0.301 (0.108)	0.312 (0.107)	0.289 (0.102)	0.14
	Pericyte	0.021 (0.026)	0.021 (0.030)	0.023 (0.024)	0.025 (0.027)	0.03 (0.028)	0.04 (0.041)	<0.001
	Satellite	0.064 (0.052)	0.072 (0.060)	0.064 (0.054)	0.058 (0.048)	0.053 (0.042)	0.067 (0.047)	0.008
	SMC	0.111 (0.031)	0.109 (0.027)	0.11 (0.031)	0.116 (0.037)	0.108 (0.042)	0.104 (0.035)	0.25
**Left Ventricle**	Adipocytes	0.078 (0.020)	0.066 (0.015)	0.071 (0.032)	0.073 (0.037)	0.067 (0.030)	0.051 (0.062)	0.076
	Endothelial	0.10 (0.034)	0.107 (0.050)	0.109 (0.074)	0.105 (0.057)	0.122 (0.103)	0.19 (0.265)	0.001
	Fibroblast	0.054 (0.061)	0.075 (0.067)	0.074 (0.086)	0.082 (0.103)	0.119 (0.125)	0.205 (0.206)	0.001
	Lymphoid	0.088 (0.025)	0.088 (0.025)	0.085 (0.025)	0.086 (0.027)	0.085 (0.030)	0.076 (0.045)	0.32
	Myeloid	0.086 (0.040)	0.072 (0.032)	0.073 (0.035)	0.071 (0.033)	0.072 (0.040)	0.073 (0.032)	0.31
	Neuronal	0.072 (0.030)	0.055 (0.034)	0.061 (0.037)	0.058 (0.040)	0.05 (0.039)	0.038 (0.054)	0.01
	Pericytes	0.053 (0.026)	0.064 (0.043)	0.055 (0.046)	0.058 (0.044)	0.063 (0.069)	0.08 (0.084)	0.31
	SMC	0.145 (0.068)	0.139 (0.053)	0.142 (0.056)	0.145 (0.059)	0.139 (0.059)	0.139 (0.064)	0.96
	VC	0.299 (0.082)	0.313 (0.128)	0.282 (0.185)	0.282 (0.187)	0.21 (0.194)	0.115 (0.285)	<0.001
**Right Atrium**	Adipocytes	0.069 (0.045)	0.061 (0.025)	0.051 (0.028)	0.061 (0.032)	0.055 (0.027)	0.057 (0.029)	0.52
	AC	0.235 (0.072)	0.265 (0.110)	0.233 (0.143)	0.211 (0.116)	0.204 (0.110)	0.221 (0.151)	0.18
	Endothelial	0.133 (0.029)	0.124 (0.029)	0.137 (0.038)	0.125 (0.048)	0.126 (0.047)	0.124 (0.054)	0.52
	Fibroblast	0.133 (0.121)	0.122 (0.093)	0.152 (0.139)	0.152 (0.109)	0.156 (0.119)	0.177 (0.140)	0.49
	Lymphoid	0.064 (0.020)	0.07 (0.024)	0.069 (0.025)	0.071 (0.028)	0.074 (0.034)	0.071 (0.035)	0.52
	Myeloid	0.104 (0.059)	0.093 (0.040)	0.088 (0.033)	0.089 (0.044)	0.085 (0.036)	0.085 (0.037)	0.85
	Neuronal	0.063 (0.025)	0.069 (0.024)	0.06 (0.032)	0.066 (0.028)	0.065 (0.027)	0.069 (0.024)	0.52
	Pericytes	0.079 (0.044)	0.086 (0.048)	0.086 (0.038)	0.088 (0.049)	0.095 (0.054)	0.083 (0.060)	0.62
	SMC	0.092 (0.070)	0.101 (0.050)	0.095 (0.058)	0.103 (0.065)	0.103 (0.055)	0.094 (0.055)	0.84

Legend: Median proportions of each cell lineage per age category with FDR-adjusted Kruskal-Wallis test of statistical significance across age categories. SMC = Smooth Muscle Cell, VC = Ventricular Cardiomyocyte, AC = Atrial Cardiomyocyte. Data presented as proportion (IQR).

**Table 4:** Age differences in lineage composition of myocardium and skeletal muscle in GTEx study defined using BayesPrism.

		20–29	30–39	40–49	50–59	60–69	70–79	FDR
**Skeletal Muscle**	Endothelial	0.049 (0.014)	0.047 (0.017)	0.049 (0.014)	0.047 (0.018)	0.047 (0.020)	0.053 (0.024)	0.36
	Fibroblast	0.014 (0.021)	0.020 (0.032)	0.020 (0.022)	0.019 (0.023)	0.026 (0.031)	0.036 (0.049)	<0.001
	Lymphoid	0.001 (0.001)	0.001 (0.001)	0.001 (0.002)	0.001 (0.002)	0.002 (0.002)	0.002 (0.002)	0.005
	Myeloid	0.004 (0.006)	0.004 (0.005)	0.004 (0.006)	0.004 (0.005)	0.003 (0.005)	0.004 (0.005)	0.52
	Myocyte	0.861 (0.049)	0.855 (0.060)	0.857 (0.055)	0.861 (0.062)	0.850 (0.070)	0.823 (0.104)	0.09
	Pericyte	0.015 (0.007)	0.015 (0.010)	0.016 (0.008)	0.017 (0.009)	0.018 (0.011)	0.020 (0.015)	0.005
	Satellite	0.023 (0.038)	0.023 (0.034)	0.017 (0.035)	0.014 (0.034)	0.008 (0.035)	0.012 (0.033)	0.005
	SMC	0.024 (0.008)	0.026 (0.010)	0.023 (0.010)	0.025 (0.012)	0.025 (0.013)	0.024 (0.015)	0.36
**Left Ventricle**	Adipocytes	<0.001 (0.004)	<0.001 (0.001)	0.001 (0.003)	0.001 (0.002)	0.001 (0.003)	0.001 (0.004)	0.131
	Endothelial	0.144 (0.046)	0.155 (0.048)	0.155 (0.090)	0.153 (0.075)	0.185 (0.136)	0.312 (0.283)	0.007
	Fibroblast	0.029 (0.031)	0.033 (0.046)	0.045 (0.067)	0.042 (0.076)	0.070 (0.102)	0.144 (0.159)	0.004
	Lymphoid	0.011 (0.023)	0.011 (0.030)	0.009 (0.024)	0.010 (0.028)	0.009 (0.029)	0.007 (0.018)	0.294
	Myeloid	0.023 (0.027)	0.015 (0.020)	0.020 (0.019)	0.016 (0.019)	0.015 (0.021)	0.017 (0.019)	0.628
	Neuronal	<0.001 (0.000)	<0.001 (0.002)	<0.001 (0.004)	<0.001 (0.007)	<0.001 (0.013)	<0.001 (0.055)	0.012
	Pericytes	<0.001 (0.006)	0.010 (0.060)	0.010 (0.088)	0.025 (0.102)	0.039 (0.146)	0.080 (0.142)	0.008
	SMC	0.240 (0.053)	0.228 (0.053)	0.222 (0.091)	0.223 (0.068)	0.197 (0.117)	0.193 (0.207)	0.008
	VC	0.525 (0.106)	0.515 (0.140)	0.500 (0.233)	0.511 (0.229)	0.407 (0.270)	0.232 (0.488)	0.004
**Right Atrium**	Adipocytes	0.003 (0.008)	<0.001 (0.003)	<0.001 (0.002)	<0.001 (0.003)	<0.001 (0.001)	<0.001 (0.002)	0.129
	AC	0.338 (0.095)	0.373 (0.124)	0.329 (0.166)	0.326 (0.141)	0.326 (0.126)	0.343 (0.154)	0.441
	Endothelia	0.253 (0.078)	0.244 (0.062)	0.249 (0.060)	0.244 (0.053)	0.247 (0.048)	0.237 (0.064)	0.837
	Fibroblast	0.125 (0.090)	0.112 (0.091)	0.151 (0.121)	0.149 (0.115)	0.157 (0.110)	0.161 (0.120)	0.441
	Lymphoid	0.003 (0.012)	0.011 (0.015)	0.006 (0.014)	0.007 (0.016)	0.008 (0.017)	0.008 (0.019)	0.658
	Myeloid	0.047 (0.030)	0.031 (0.035)	0.027 (0.024)	0.027 (0.030)	0.025 (0.030)	0.0191 (0.024)	0.441
	Neuronal	0.002 (0.004)	0.004 (0.008)	0.002 (0.006)	0.003 (0.006)	0.002 (0.007)	0.002 (0.007)	0.807
	Pericytes	<0.001 (0.023)	<0.001 (0.001)	<0.001 (0.023)	<0.001 (0.031)	<0.001 (0.049)	<0.001 (0.048)	0.193
	SMC	0.338 (0.095)	0.373 (0.1239)	0.329 (0.1656)	0.326 (0.1407)	0.326 (0.126)	0.343 (0.154)	0.658

Legend: Median proportions of each cell lineage per age category with FDR-adjusted Kruskal-Wallis test of statistical significance across age categories. SMC = Smooth Muscle Cell, VC = Ventricular Cardiomyocyte, AC = Atrial Cardiomyocyte. Data presented as proportion (IQR).

**Table 5:** Sex differences in lineage composition of myocardium and skeletal muscle using CIBERSORTx or BayesPrism in the GTEx study.

		CIBERSORTx			BayesPrism		
		Male	Female	FDR	Male	Female	FDR
**Skeletal Muscle**	Endothelial	0.129 (0.036)	0.129 (0.032)	0.41	0.048 (0.018)	0.049 (0.017)	0.21
	Fibroblast	0.094 (0.044)	0.096 (0.044)	0.77	0.020 (0.26)	0.024 (0.028)	0.051
	Lymphoid	0.13 (0.053)	0.128 (0.053)	0.77	0.001 (0.001)	0.001 (0.002)	0.018
	Myeloid	0.107 (0.072)	0.124 (0.059)	0.005	0.003 (0.005)	0.004 (0.005)	0.047
	Myocyte	0.308 (0.105)	0.291 (0.083)	0.005	0.861 (0.063)	0.850 (0.062)	0.007
	Pericyte	0.026 (0.029)	0.026 (0.052)	0.77	0.017 (0.009)	0.016 (0.010)	0.08
	Satellite	0.056 (0.047)	0.066 (0.053)	0.005	0.012 (0.033)	0.021 (0.037)	0.002
	SMC	0.11 (0.036)	0.114 (0.035)	0.77	0.024 (0.011)	0.025 (0.012)	0.94
**Left Ventricle**	Adipocytes	0.068 (0.036)	0.073 (0.026)	0.052	<0.001 (0.002)	<0.001 (0.004)	0.28
	Endothelial	0.113 (0.089)	0.108 (0.054)	0.18	0.161 (0.125)	0.158 (0.078)	0.16
	Fibroblast	0.105 (0.139)	0.072 (0.069)	0.011	0.057 (0.104)	0.043 (0.061)	0.049
	Lymphoid	0.085 (0.029)	0.09 (0.024)	0.061	0.019 (0.018)	0.014 (0.014)	0.02
	Myeloid	0.072 (0.035)	0.076 (0.041)	0.11	0.015 (0.020)	0.021 (0.018)	0.03
	Neuronal	0.052 (0.036)	0.066 (0.035)	<0.001	<0.001 (0.011)	<0.001 (0.004)	0.16
	Pericytes	0.062 (0.060)	0.056 (0.038)	0.18	0.021 (0.119)	0.004 (0.074)	0.08
	SMC	0.143 (0.062)	0.139 (0.050)	0.66	0.209 (0.102)	0.223 (0.086)	0.03
	VC	0.259 (0.199)	0.281 (0.178)	0.12	0.469 (0.259)	0.506 (0.181)	0.049
**Right Atrium**	Adipocytes	0.055 (0.028)	0.062 (0.032)	0.001	<0.001 (0.001)	<0.001 (0.006)	0.006
	AC	0.207 (0.116)	0.233 (0.102)	0.017	0.317 (0.131)	0.344 (0.118)	0.006
	Endothelial	0.127 (0.048)	0.126 (0.034)	0.96	0.246 (0.055)	0.245 (0.051)	0.96
	Fibroblast	0.166 (0.114)	0.14 (0.113)	0.019	0.162 (0.144)	0.122 (0.088)	0.002
	Lymphoid	0.075 (0.028)	0.064 (0.031)	<0.001	0.008 (0.016)	0.006 (0.014)	0.14
	Myeloid	0.085 (0.043)	0.092 (0.034)	0.038	0.025 (0.032)	0.029 (0.027)	0.24
	Neuronal	0.066 (0.028)	0.063 (0.023)	0.91	0.003 (0.007)	0.002 (0.005)	0.74
	Pericytes	0.095 (0.052)	0.085 (0.039)	0.085	<0.001 (0.044)	<0.001 (0.016)	0.002
	SMC	0.102 (0.062)	0.097 (0.055)	0.91	0.204 (0.062)	0.209 (0.048)	0.17

Legend: Median proportions of each cell lineage per sex with FDR-adjusted Mann-Whitney test of statistical significance across sex. SMC = Smooth Muscle Cell, VC = Ventricular Cardiomyocyte, AC = Atrial Cardiomyocyte. Data presented as proportion (IQR).

With regard to LV myocardium, CIBERSORTx indicated marked increases in the proportions of endothelial cells and fibroblasts with advancing age, mirrored by decreasing proportions of neuronal cells and ventricular cardiomyocytes (Table 3; Supplementary Fig. 8). However, in RA myocardium, CIBERSORTx found no statistically significant differences associated with age (Table 3; Supplementary Fig. 9). BayesPrism concurred with CIBERSORTx-identified compositional differences in LV myocardium, except for neuronal cells which it predicted to increase with old age; unlike CIBERSORTx, it also identified rising pericyte proportion and declining vascular smooth muscle cell proportion with advancing age (Table 4 and Supplementary Fig. 10). For RA myocardium, BayesPrism fully concurred with CIBERSORTx that there were no significant differences in lineage composition with age (Table 4 and Supplementary Fig. 11). Notably, BayesPrism suggested higher proportions of endothelial, vascular smooth muscle and cardiomyocytes lineages than CIBERSORTx, accompanied by lower estimated proportions of other lineages. As illustrated in Supplementary Fig. 12 for LV myocardium, concordance between CIBERSORTx and Bayes Prism was poor for some lineages (e.g. adipocyte R^2^ = 0.1) and excellent for others (e.g. endothelial cell R^2^ = 0.94). As illustrated in Supplementary Fig. 13 for RA myocardium, concordance between CIBERSORTx and Bayes Prism was modest to strong, ranging from R^2^ of 0.4 for vascular smooth muscle cells to 0.91 for atrial cardiomyocytes.

In LV samples from women, CIBERSORTx indicated a higher proportion of neuronal cells and a lower proportion of fibroblasts (Table 5 and Supplementary Fig. 14). BayesPrism concurred with the lower proportion of fibroblasts in women, but did not find a difference in neuronal content; unlike CIBRSORTx, it also found lower lymphoid and higher myeloid, vascular smooth muscle and cardiomyocyte content (Table 5 and Supplementary Fig. 15). Interestingly, CIBERSORTx also found a lower proportion of fibroblasts in RA samples from women, although neuronal content was not different to men; it also found that women had a higher proportion of adipocytes, myeloid and cardiomyocytes, and lower proportions of lymphoid cells in RA (Table 5 and Supplementary Fig. 16). BayesPrism concurred in these findings for fibroblasts, cardiomyocytes and adipocytes, but did not demonstrate differences in lymphoid or myeloid cells (Table 5 and Supplementary Fig. 17); it also found a lower proportion of pericytes in women, although these were a rare population (predicted at around 0.1 % of cells). As a sensitivity analysis, we compared BayesPrism outputs when trained using the 200 cells per lineage used in our main analyses, versus all Heart Cell Atlas right atrial cells; this revealed excellent agreement with R2 values of >0.95 for all lineages except pericytes with a value of 0.84 (Supplemental Fig. 18). This suggests that using a larger quantity of training data does not substantially influence our BayesPrism estimates.

## 4 Discussion

Determining the cellular composition of skeletal muscle and myocardial tissue samples is a complex and resource-intensive task to perform at scale, whether by histology, flow cytometry or single cell/nucleus RNA-seq. We present alternative methods to rapidly define the cellular composition of these tissues from existing or newly collected bulk RNA-seq data using either the CIBERSORTx or BayesPrism deconvolution packages [6,12]. We show that these approaches are robust *in silico* and yield expected differences in cell lineages that are known to change with ageing in skeletal muscle. Moreover, we use our pipeline to show how age and sex are associated with differing composition in RA and LV myocardium, showing its potential to highlight cell lineages that warrant further exploration in future research exploring the biology of ageing- or sex-differences. By sharing our methods and analyses, other scientists are now able to apply our validated approach to their own data to learn about the composition of the skeletal muscle and myocardial samples they are studying. Whilst deconvolution approaches are widely used in immunology and cancer research, this is not the case in myocardial and skeletal muscle research. Our data now suggest that deconvolution can provide additional insights from myocardial and skeletal muscle bulk RNA-seq data, highlighting cell lineages that warrant further exploration in studies of physiology and disease.

Notably, at the level of individual samples, BayesPrism and CIBERSORTx demonstrated weak correlation for some cell lineages and strong correlation for others, despite using the same training single cell/nucleus RNA-seq data, likely reflecting their differing analytical methods. Moreover, even in cases where they correlated well, they often produced substantially different estimates of lineage proportions; for example, ventricular cardiomyocyte estimates correlated robustly (R^2^ = 0.94), but BayesPrism yielded systematically higher proportion estimates. In general, lineage proportion estimates from CIBERSORTx were closer to those seen in the Heart Cell Atlas training data, but unclear if those proportions are reflective of those in the GTEx cohort samples. This emphasises a need for further validation studies using samples with paired bulk RNA-seq deconvolution and other estimates of tissue composition e.g. from single nucleus RNA-seq or immunohistochemistry. Until such a validation of each approach, it is important to use their inferred absolute cell lineage estimates with caution. However, it is reassuring that qualitative predictions of age-sex differences in skeletal muscle, right atrium and left ventricle using CIBERSORTx and BayesPrism often concurred, with 13/20 (65 %) CIBERSORTx hits and 13/24 (54 %) BayesPrism hits corroborated by the other method. This suggests that it is reasonable to use either approach to infer relative between-group differences in tissue composition, until evidence from alternate methods (such as immuno-histochemistry) is able to determine if one approach is superior.

We show that ageing is associated with wide-ranging differences in skeletal muscle composition. These may relate to a phenomenon called sarcopenia, which denotes a progressive loss of muscle mass and function, often associated with ageing [18], and is linked to higher rates of mortality and hospitalisation [19]. The decrease in satellite cells we observed with both deconvolution packages between 20-29 and 60–69 years of age is typical of ageing, having previously been demonstrated in mice [20,21]. These are multipotent cells that can rejuvenate myocytes, and our observation that they broadly decrease with age supports the validity of our deconvolution process. Whilst our data do not show this trend continuing at 70–79 years of age, this group is substantially smaller than other age groups in our study and so should be interpreted cautiously. The increased fibroblast preponderance identified by both deconvolution methods in aged muscle is also not unexpected, as satellite cells become more fibrogenic with age in a process regulated by Wnt signalling [22]. Whilst the age-associations we describe in other skeletal muscle cell lineages are less well explored, a small-scale scRNA-seq/snRNA-seq study supports our observation that endothelial cell and pericyte populations may increase with age [23]. Importantly, these data show that some lymphoid populations increase with age, in contrast with our CIBERSORTx results indicating a lower proportion of total lymphoid cells, although BayesPrism noted an increasing proportion with age. This indicates the need for further replication studies, but also emphasises that our data may not reflect trends in important subsets of the lineages we define. It is also notable that our approach defines the proportional cell lineage contribution to RNA molecules, rather than directly estimating the proportion of cells. This means our approach may emphasise differences arising from varying proportions of cell types with the greatest RNA content. However, our approach is a useful method to assess potential relative changes in cell linages in large cohorts, which can be validated and extended with alternative approaches in a subset of samples. Indeed, our data suggest that ageing is associated with wide-ranging differences in skeletal muscle cell lineage composition that warrant further, more detailed, exploration.

Ageing-associated differences in myocardial composition are also of great potential importance; for example, the clinical syndrome of heart failure affects more than 64 million people globally, and is increasingly prevalent as populations live longer [24]. Notably, scRNA-seq has demonstrated fewer cardiomyocytes and more endothelial cells in LV myocardium from people with heart failure due to ischemic heart disease [25]. Lower cardiomyocyte content has also been described in LV myocardial snRNA-seq data from people with dilated or hypertrophic cardiomyopathy versus controls [26]. We saw a striking reduction in ventricular, but not atrial, cardiomyocyte content with increasing age using both deconvolution methods; we do not know whether these older groups had more prevalent heart failure or other myocardial disease, although this would be a reasonable supposition. We also observed higher LV myocardial fibroblast content with increasing age using both deconvolution methods, which is a well-recognised phenomenon associated with myocardial stiffness [27], although it is interesting that this was not mirrored in the RA myocardium. Indeed, we observed no substantial differences in lineage composition with age in the RA myocardium, perhaps suggesting that ageing has differential impacts on the RA versus the LV. This has broad implications, suggesting that findings from one region of the heart may not generalise to other regions. Other differences in the LV as age increased, including higher endothelial cell content, whilst of smaller absolute magnitude, are also of potential relevance to ageing and warrant further exploration to define their physiological importance.

With regard to sex-differences in skeletal muscle composition, using both deconvolution methods we observed modestly greater proportions of satellite and myeloid cell proportions in women, whilst their myocyte proportion was lower. Data from animal studies, in particular, support the capacity of oestrogens to modulate satellite cells and so this finding is plausible [28], although large scale human studies are needed to validate and extend this finding and the other lineage differences we observed. In LV myocardium, we observed women to have a lower fibroblast content using both deconvolution methods, whilst only CIBERSORTx found a higher neuronal content. The former is expected based on a known lower prevalence of magnetic resonance imaging-defined myocardial fibrosis in women [29], whilst there is also clinical evidence of sexual dimorphism in cardiac neuronal activity based on MIBG scintigraphy [30]. Similar to the LV, using both deconvolution methods we noted that women also had a lower fibroblast content in the RA. However, as noted for our age data, sex differences in the LV myocardium were generally not reflected by those in the RA, where we noted lower lymphoid and higher myeloid, adipocyte and atrial cardiomyocyte content in women, although only the latter two were corroborated by BayesPrism. We are unaware of published data addressing sex-differences in the cellular composition of atrial myocardium and so our novel findings warrant further exploration using complementary approaches.

Whilst our study has strengths, outlined above, it is also important to discuss its limitations. First, both deconvolution pipelines used data from the Heart Cell Atlas, which provides a detailed and technically robust assessment of data from 14 human donors in multiple heart regions. These people were organ donors without a history of heart disease, with a narrow age range and likely preponderance of European ethnicity. Therefore, it is possible that our approach will perform less well in other populations, especially in disease contexts where gene expression patterns in major cell lineages may quantitatively differ, or where other unrepresented cell types may be present in the sample. For example, we excluded myocardial mesothelial cells as their low abundance (0.1 % of cells in Heart Cell Atlas) precluded inclusion and independent *in silico* validation; deconvolution of samples with much higher proportions of this lineage could lead to less accurate outputs. Whilst we have shown that our approach is robust to extremes of cellular composition in samples, it is important to be more cautious in studying data arising from circumstances that widely differ from the Heart Cell Atlas donors. However, until single cell/nucleus RNA-seq data from more diverse populations become available, we are unable to address this limitation. It is also possible to generate signature matrices in more tightly defined subgroups (e.g. females), which may improve deconvolution accuracy in these subgroups, but such data are difficult to compare with parallel data arising from other subgroups (e.g. males), hence we did not pursue this approach. Second, as noted above, it is important to interpret the outputs of CIBERSORTx as proportions of total sample RNA arising from cell lineages, rather than as proportions of cells in the samples. This is because different cell types will have different RNA contents, and therefore make lower or higher contributions to total RNA than expected based in their cellular abundance. This means that data are most appropriately compared within cell lineages to define relative differences between groups. Finally, whilst we have sought to compare our age- and sex-difference data with published work using different methodologies, often these data are not available from large human studies and so true external validation will require extensive follow-on studies. However, our comparison of two deconvolution pipelines led to agreement in 13/20 65 % of CIBERSORTx identified age-sex differences and 13/24 (54 %) BayesPrism identified age-sex differences, which increases confidence in these hits. Moreover, further validation of our pipelines is required to fully assess their performance, preferably using paired bulk and single cell/nucleus RNA-seq in the samples, possibly accompanied by orthogonal approaches like immunohistochemistry. We are unaware of suitable datasets to perform such experiments, which are the gold standard in validation of RNA-seq deconvolution methods, and so generation of these is an important future research goal. This limitation also led us not to pursue inferred gene expression profiles within lineages, an approach supported by both CIBERSORTx and BayesPrism, since we lack data with paired bulk RNA-seq and single cell/nucleus RNA-seq to validate the wealth of derived data. Future studies should pursue this exciting possibility once suitable data become available.

In summary, we have derived and validated two approaches that allow inference of the cellular composition of human skeletal muscle, RA myocardium and LV myocardium samples based on their bulk transcriptome. Our pipelines can be easily and rapidly applied by the scientific community to derive additional insights from existing or emerging bulk RNA-seq data. By defining the most clinically and statistically significant differences in the lineage composition of these samples, subsequent studies focussing on these lineages can be made less costly and use precious human biopsies more efficiently. This approach has the potential to expedite the study of human biology and disease, with potential value in a wide range of applications.

## CRediT authorship contribution statement

**Marcella Conning-Rowland:** Writing – original draft, Investigation, Formal analysis, Data curation, Conceptualization. **Chew W. Cheng:** Writing – review & editing. **Oliver Brown:** Writing – review & editing. **Marilena Giannoudi:** Writing – review & editing. **Eylem Levelt:** Supervision. **Lee D. Roberts:** Writing – review & editing, Supervision. **Kathryn J. Griffin:** Supervision. **Richard M. Cubbon:** Writing – review & editing, Writing – original draft, Supervision, Conceptualization.

## Ethics statement

Ethical approval was not required for this study. Approvals required for the publicly available source data are provided by the Heart Cell Atlas (https://www.heartcellatlas.org) and GTEx project (https://www.gtexportal.org) websites.

## Disclosures

None.

## Data availability statement

Raw data used in this project are publicly available from the Heart Cell Atlas (https://www.heartcellatlas.org) and GTEx project (https://www.gtexportal.org). Details of the operating systems, code and workflows used are provided in the methods section, along with supplementary files 10 and 11.

## Funding

British Heart Foundation (BHF) (FS/4yPhD/F/21/34153).

## Declaration of competing interest

The authors declare the following financial interests/personal relationships which may be considered as potential competing interests: Richard Cubbon reports a relationship with Janssen Oncology that includes: speaking and lecture fees. Oliver Brown reports a relationship with Novartis that includes: speaking and lecture fees. If there are other authors, they declare that they have no known competing financial interests or personal relationships that could have appeared to influence the work reported in this paper.

## References

[1] Jovic D., Liang X., Zeng H., Lin L., Xu F., Luo Y. (2022). Single-cell RNA sequencing technologies and applications: a brief overview. Clin. Transl. Med..

[2] Wang M., Gu M., Liu L., Liu Y., Tian L. (2021). Single-cell RNA sequencing (scRNA-seq) in cardiac tissue: applications and limitations. Vasc Heal Risk Manag.

[3] Avila Cobos F., Vandesompele J., Mestdagh P., De Preter K. (2018). Computational deconvolution of transcriptomics data from mixed cell populations. Bioinformatics.

[4] Wang X., Park J., Susztak K., Zhang N.R., Li M. (2019). Bulk tissue cell type deconvolution with multi-subject single-cell expression reference. Nat. Commun..

[5] Li B., Li T., Liu J.S., Liu X.S. (2020). Computational deconvolution of tumor-infiltrating immune components with bulk tumor gene expression data. Methods Mol. Biol..

[6] Newman A.M., Steen C.B., Liu C.L., Gentles A.J., Chaudhuri A.A., Scherer F. (2019). Determining cell type abundance and expression from bulk tissues with digital cytometry. Nat. Biotechnol..

[7] Jin H., Liu Z. (2021). A benchmark for RNA-seq deconvolution analysis under dynamic testing environments. Genome Biol..

[8] Newman A.M., Liu C.L., Green M.R., Gentles A.J., Feng W., Xu Y. (2015). Robust enumeration of cell subsets from tissue expression profiles. Nat. Methods.

[9] Chen S.H., Kuo W.Y., Su S.Y., Chung W.C., Ho J.M., Lu H.H.S. (2018). A gene profiling deconvolution approach to estimating immune cell composition from complex tissues. BMC Bioinf..

[10] Chen B., Khodadoust M.S., Liu C.L., Newman A.M., Alizadeh A.A. (2018). Profiling tumor infiltrating immune cells with CIBERSORT. Methods Mol. Biol..

[11] Steen C.B., Liu C.L., Alizadeh A.A., Newman A.M. (2020). Profiling cell type abundance and expression in bulk tissues with CIBERSORTx. Methods Mol. Biol..

[12] Chu T., Wang Z., Pe’er D., Danko C.G. (2022). Cell type and gene expression deconvolution with BayesPrism enables Bayesian integrative analysis across bulk and single-cell RNA sequencing in oncology. Nat Cancer.

[13] Cohen C.D., De Blasio M.J., Lee M.K.S., Farrugia G.E., Prakoso D., Krstevski C. (2021). Diastolic dysfunction in a pre-clinical model of diabetes is associated with changes in the cardiac non-myocyte cellular composition. Cardiovasc. Diabetol..

[14] Squiers G.T., McLellan M.A., Ilinykh A., Branca J., Rosenthal N.A., Pinto A.R. (2021). Cardiac cellularity is dependent upon biological sex and is regulated by gonadal hormones. Cardiovasc. Res..

[15] Litviňuková M., Talavera-López C., Maatz H., Reichart D., Worth C.L., Lindberg E.L. (2020). Cells of the adult human heart. Nature.

[16] Luca B.A., Steen C.B., Matusiak M., Azizi A., Varma S., Zhu C. (2021). Atlas of clinically distinct cell states and ecosystems across human solid tumors. Cell.

[17] Consortium GTEx (2020). The GTEx Consortium atlas of genetic regulatory effects across human tissues. Science..

[18] Cruz-Jentoft A.J., Sayer A.A. (2019). Sarcopenia. Lancet..

[19] Beaudart C., Zaaria M., Pasleau F., Reginster J.Y., Bruyère O. (2017). Health outcomes of sarcopenia: a systematic review and meta-analysis. PLoS One.

[20] Gibson M.C., Schultz E. (1983). Age-related differences in absolute numbers of skeletal muscle satellite cells. Muscle Nerve.

[21] Shefer G., Van de Mark D.P., Richardson J.B., Yablonka-Reuveni Z. (2006). Satellite-cell pool size does matter: defining the myogenic potency of aging skeletal muscle. Dev. Biol..

[22] Brack A.S., Conboy M.J., Roy S., Lee M., Kuo C.J., Keller C. (2007). Increased Wnt signaling during aging alters muscle stem cell fate and increases fibrosis. Science..

[23] Kedlian V.R., Wang Y., Liu T., Chen X., Bolt L., Tudor C. (2024). Human skeletal muscle aging atlas. Nat Aging..

[24] Savarese G., Becher P.M., Lund L.H., Seferovic P., Rosano G.M.C., Coats A.J.S. (2023). Global burden of heart failure: a comprehensive and updated review of epidemiology. Cardiovasc. Res..

[25] Simonson B., Chaffin M., Hill M.C., Atwa O., Guedira Y., Bhasin H. (2023). Single-nucleus RNA sequencing in ischemic cardiomyopathy reveals common transcriptional profile underlying end-stage heart failure. Cell Rep..

[26] Chaffin M., Papangeli I., Simonson B., Akkad A.-D., Hill M.C., Arduini A. (2022). Single-nucleus profiling of human dilated and hypertrophic cardiomyopathy. Nature.

[27] Trial J., Cieslik K.A. (2018). Changes in cardiac resident fibroblast physiology and phenotype in aging. Am J Physiol Hear Circ Physiol.

[28] Jomard C., Gondin J. (2023). Influence of sexual dimorphism on satellite cell regulation and inflammatory response during skeletal muscle regeneration. Physiol Rep.

[29] Chehab O., Shabani M., Varadarajan V., Wu C.O., Watson K.E., Yeboah J. (2023). Endogenous sex hormone levels and myocardial fibrosis in men and postmenopausal women. JACC Adv.

[30] Sakata K., Shirotani M., Yoshida H., Kurata C. (1998). Physiological fluctuation of the human left ventricle sympathetic nervous system assessed by iodine-123-MIBG. J. Nucl. Med..

