# Supplementary material for: Application of CIBERSORTx and BayesPrism to deconvolution of bulk RNA-seq data from human myocardium and skeletal muscle
Source: Heliyon. 2025 Feb 10;11(4):e42499. doi: 10.1016/j.heliyon.2025.e42499 (PMC11872574; doi:10.1016/j.heliyon.2025.e42499)
Supplement: Multimedia component 10 [file mmc10.docx]

**Supplementary Figure 1: In silico validation of CIBERSORTx using synthetic left ventricular tissue**
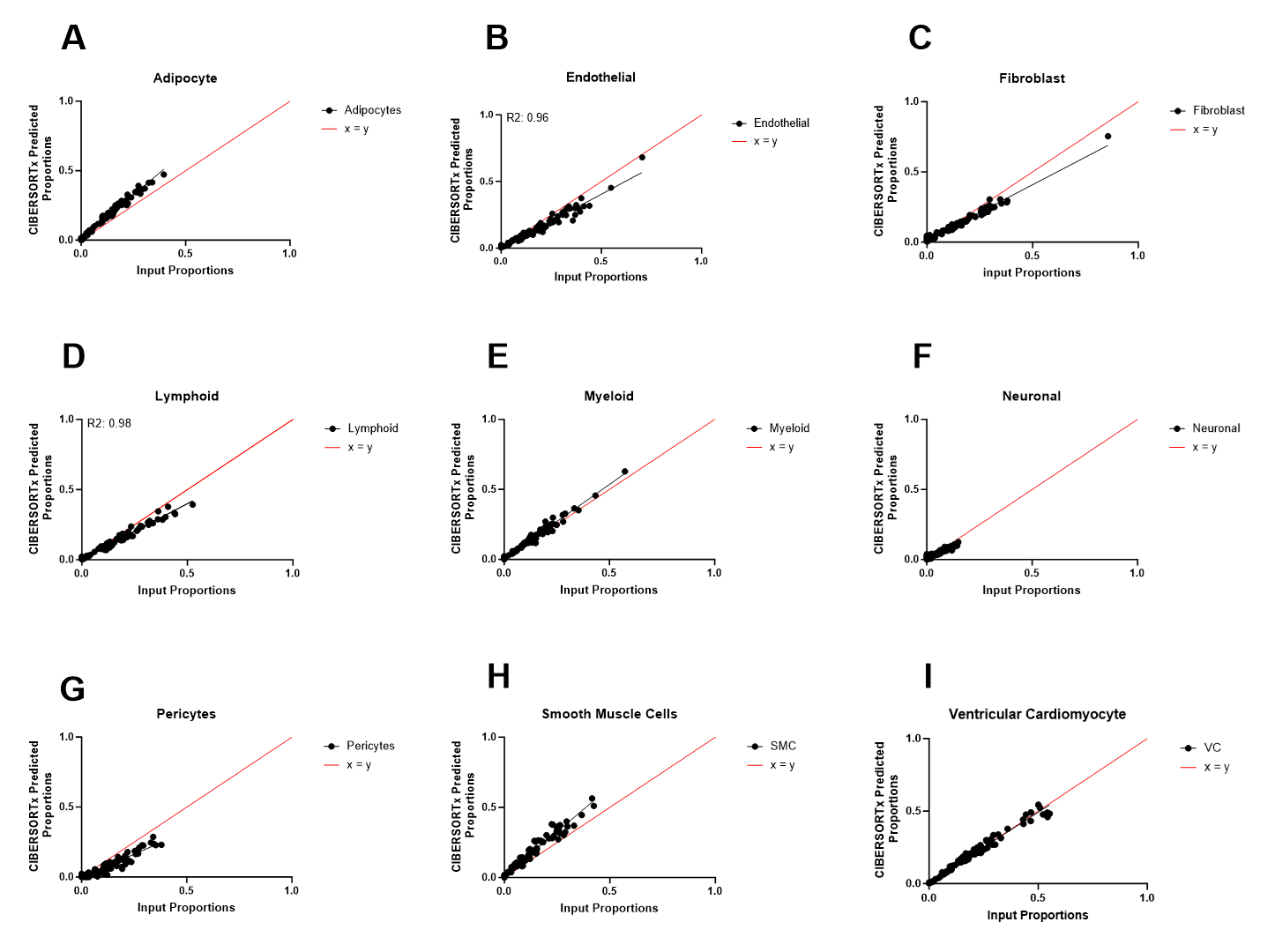


CIBERSORTx prediction of proportion of cell lineages contrasted against known cell lineage proportions (input proportions) in synthetic left ventricular tissues (n=100).

**Supplementary Figure 2: In silico validation of CIBERSORTx using synthetic right atrial tissue**


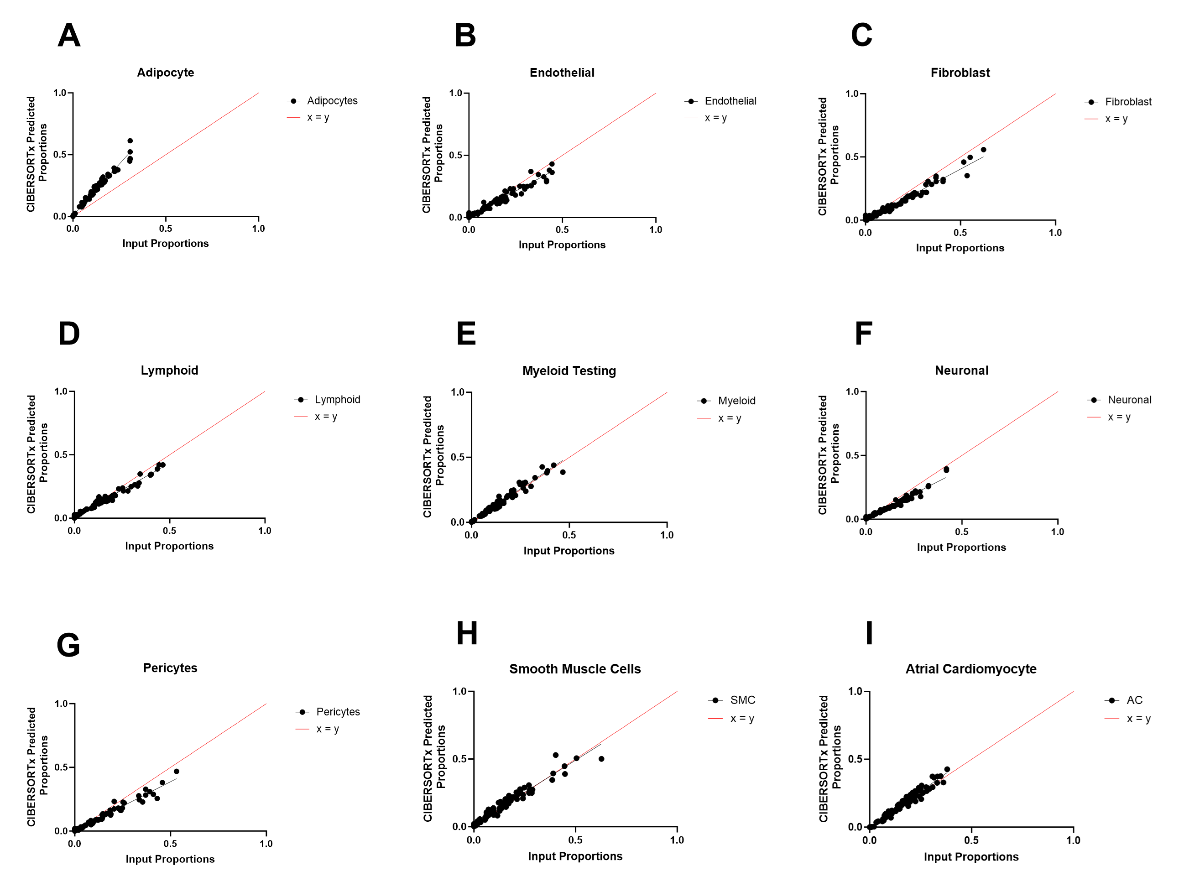


CIBERSORTx prediction of proportion of cell lineages contrasted against known cell lineage proportions (input proportions) in synthetic right atrial tissues (n=100).

**Supplementary Figure 2: In silico validation of CIBERSORTx using synthetic skeletal muscle tissue**


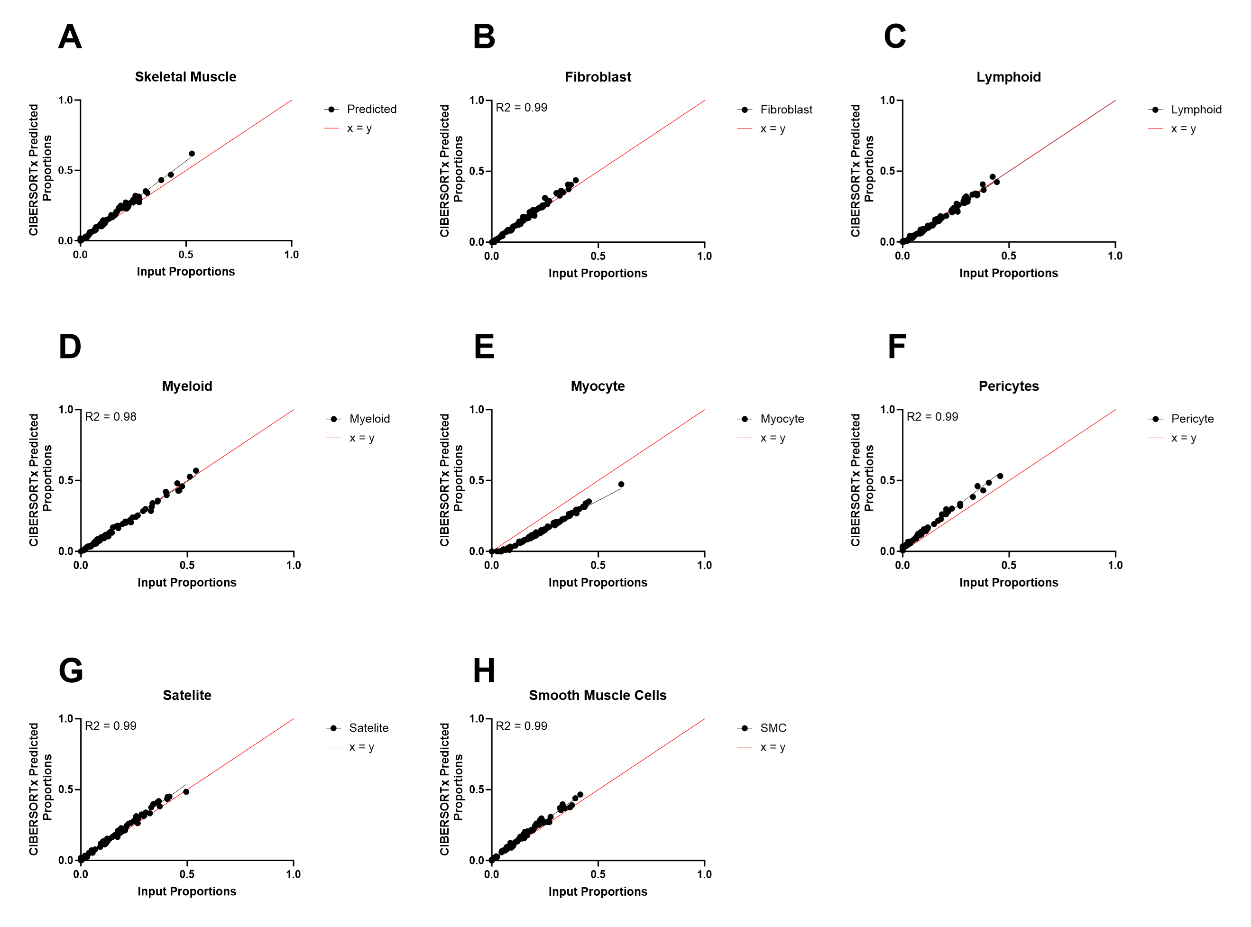


CIBERSORTx prediction of proportion of cell lineages contrasted against known cell lineage proportions (input proportions) in synthetic skeletal muscle tissues (n=100).

**Supplementary Figure 4: In silico validation of BayesPrism using synthetic left ventricular tissue**


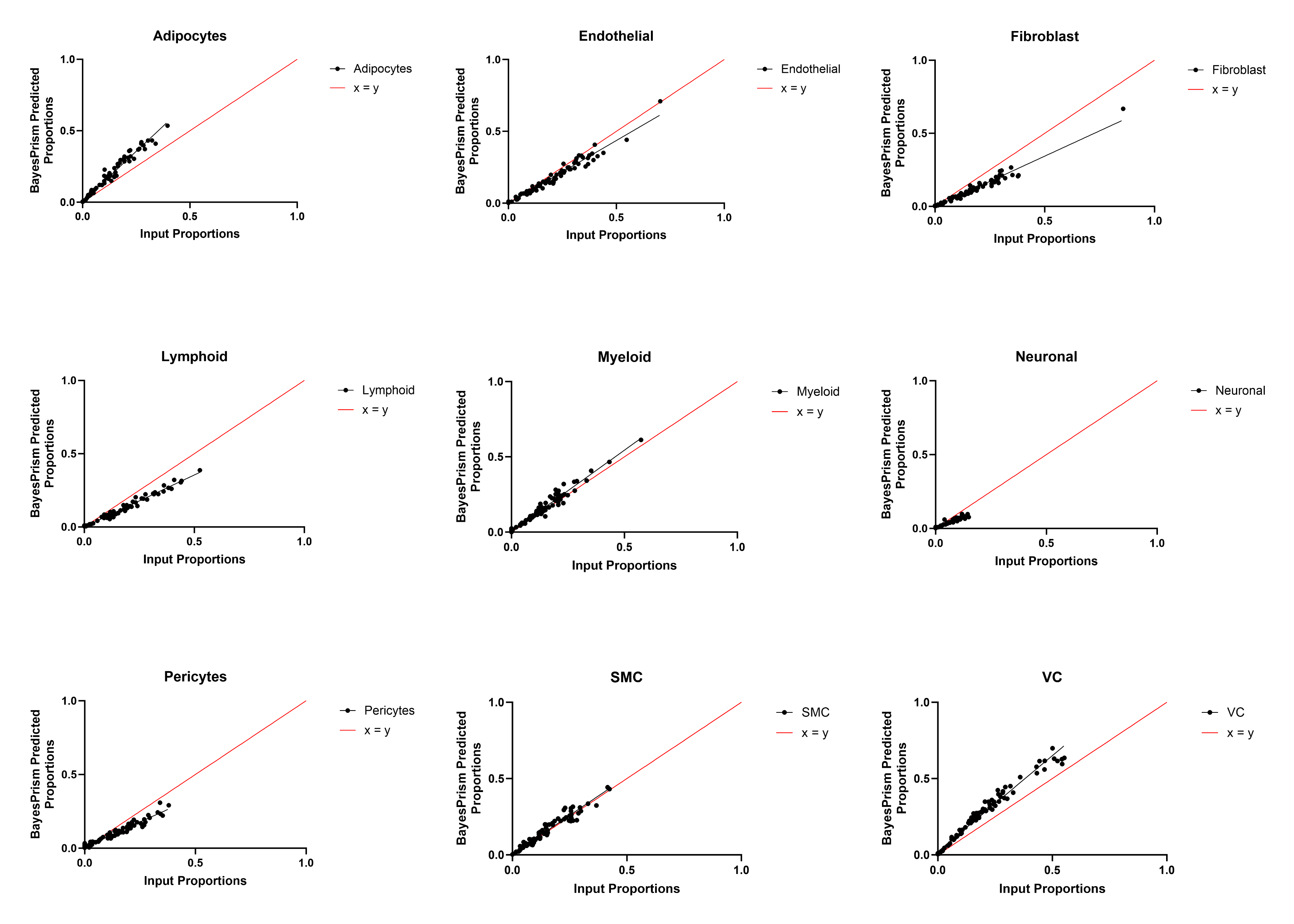


BayesPrism prediction of proportion of cell lineages contrasted against known cell lineage proportions (input proportions) in synthetic left ventricular tissues (n=100).

**Supplementary Figure 5: In silico validation of BayesPrism using synthetic right atrial tissue**


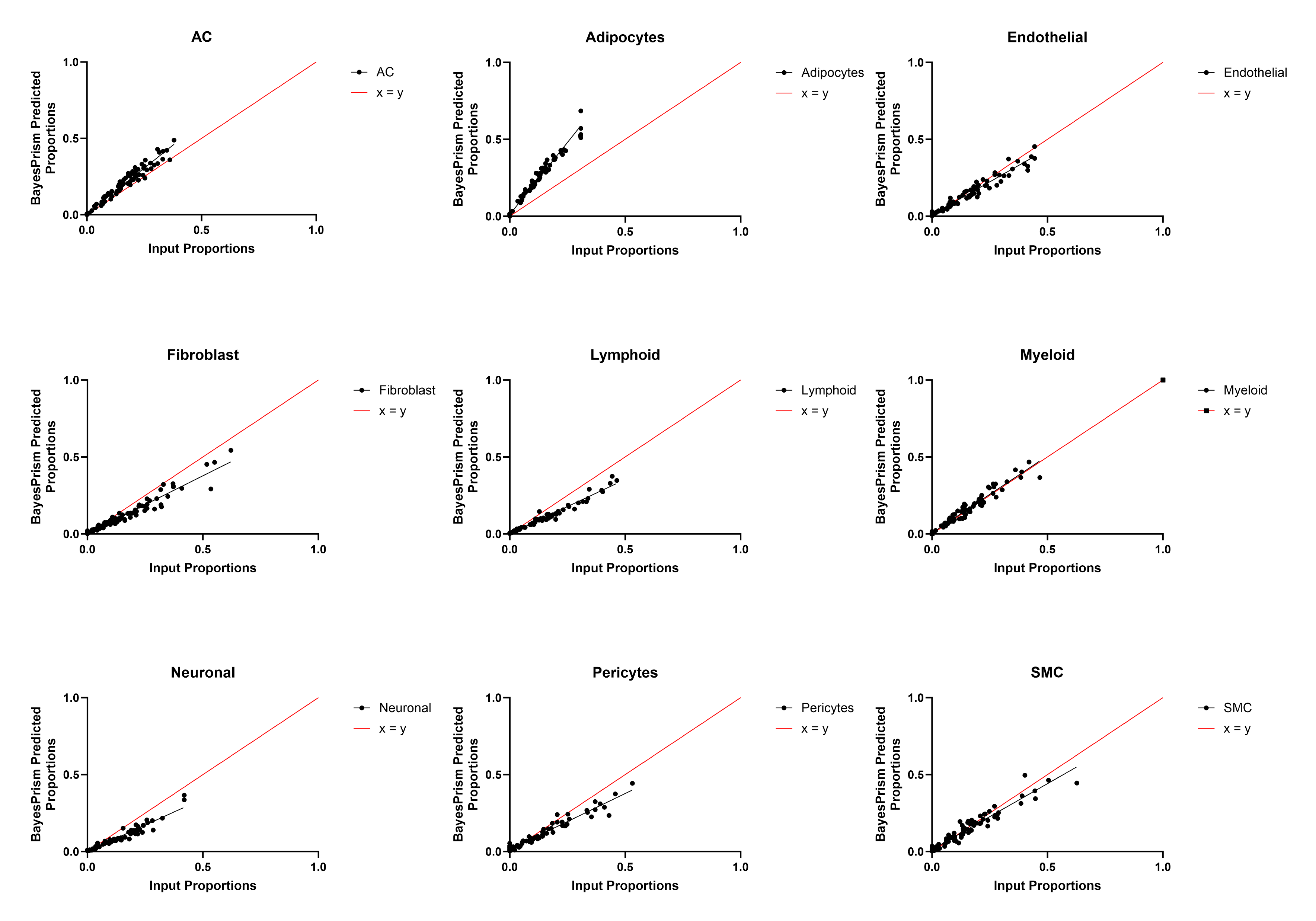


BayesPrism prediction of proportion of cell lineages contrasted against known cell lineage proportions (input proportions) in synthetic right atrial tissues (n=100).

**Supplementary Figure 6: In silico validation of BayesPrism using synthetic skeletal muscle tissue**


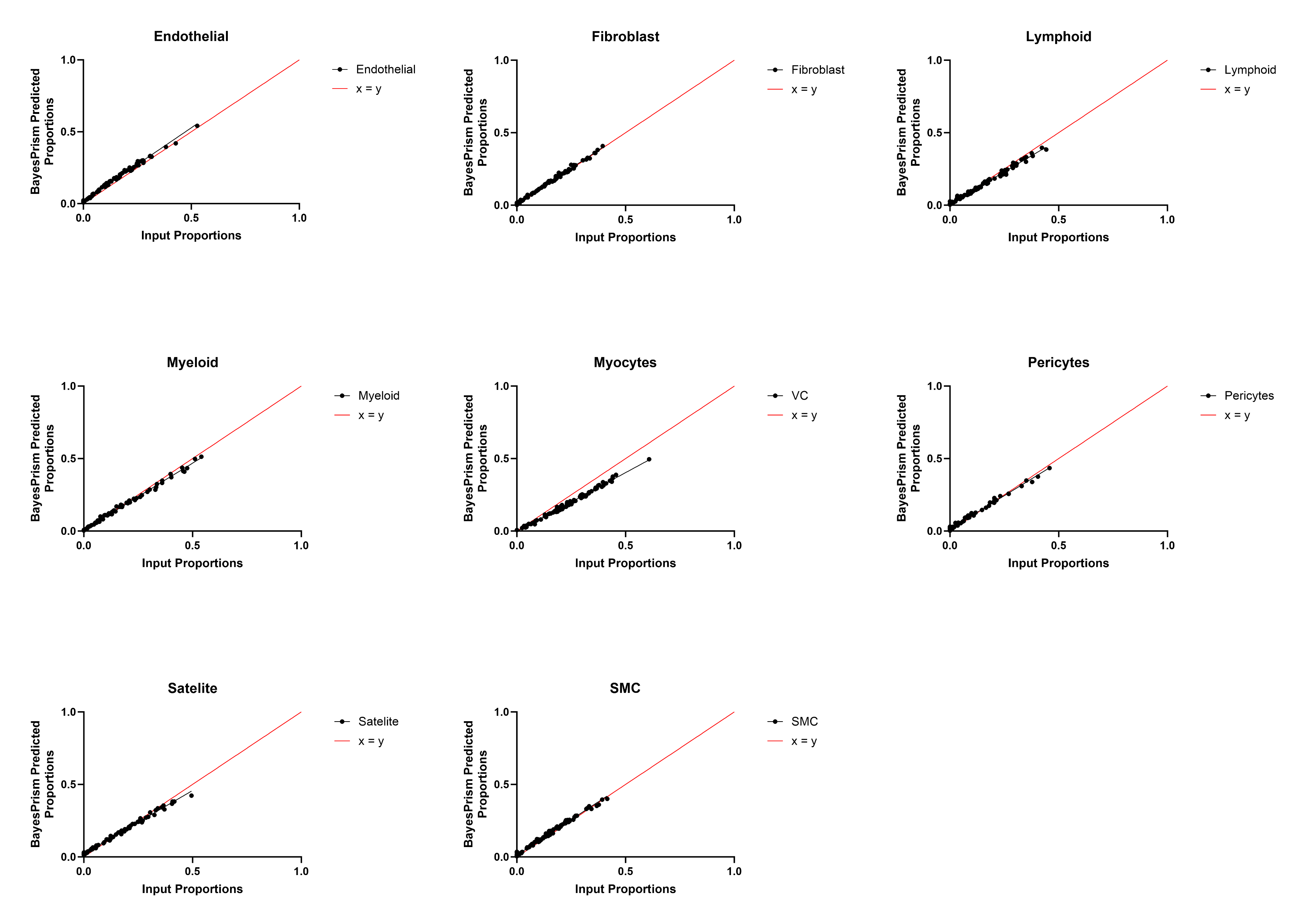


BayesPrism prediction of proportion of cell lineages contrasted against known cell lineage proportions (input proportions) in synthetic skeletal muscle tissues (n=100).

**Supplementary Figure 7: Comparison of cell lineage predictions produced by CIBERSORTx and BayesPrism using GTEx skeletal muscle tissue data**


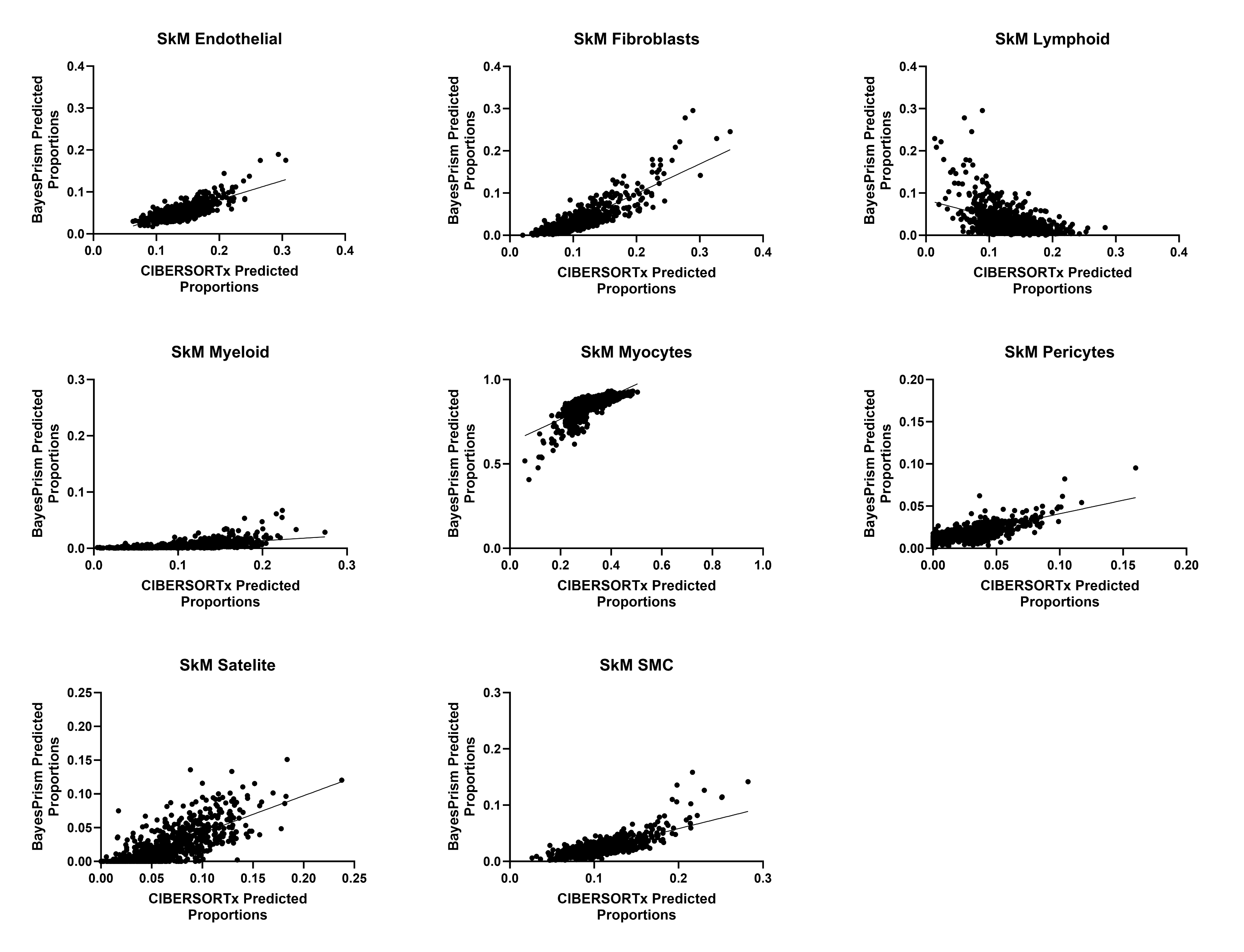


CIBERSORTx versus BayesPrism cell lineage predictions in skeletal muscle (SkM) data from GTEx (n=786). Co-efficients of determination (R^2^) for each lineage are: Endothelial = 0.58; Fibroblast = 0.76; Lymphoid = 0.21; Myeloid = 0.33; Myocyte = 0.57; Pericyte = 0.57; Satellite = 0.54; smooth muscle cell (SMC) = 0.55.

**Supplementary Figure 8: Age-associated differences in left ventricular cell lineage composition in GTEx cohort using CIBERSORTx**


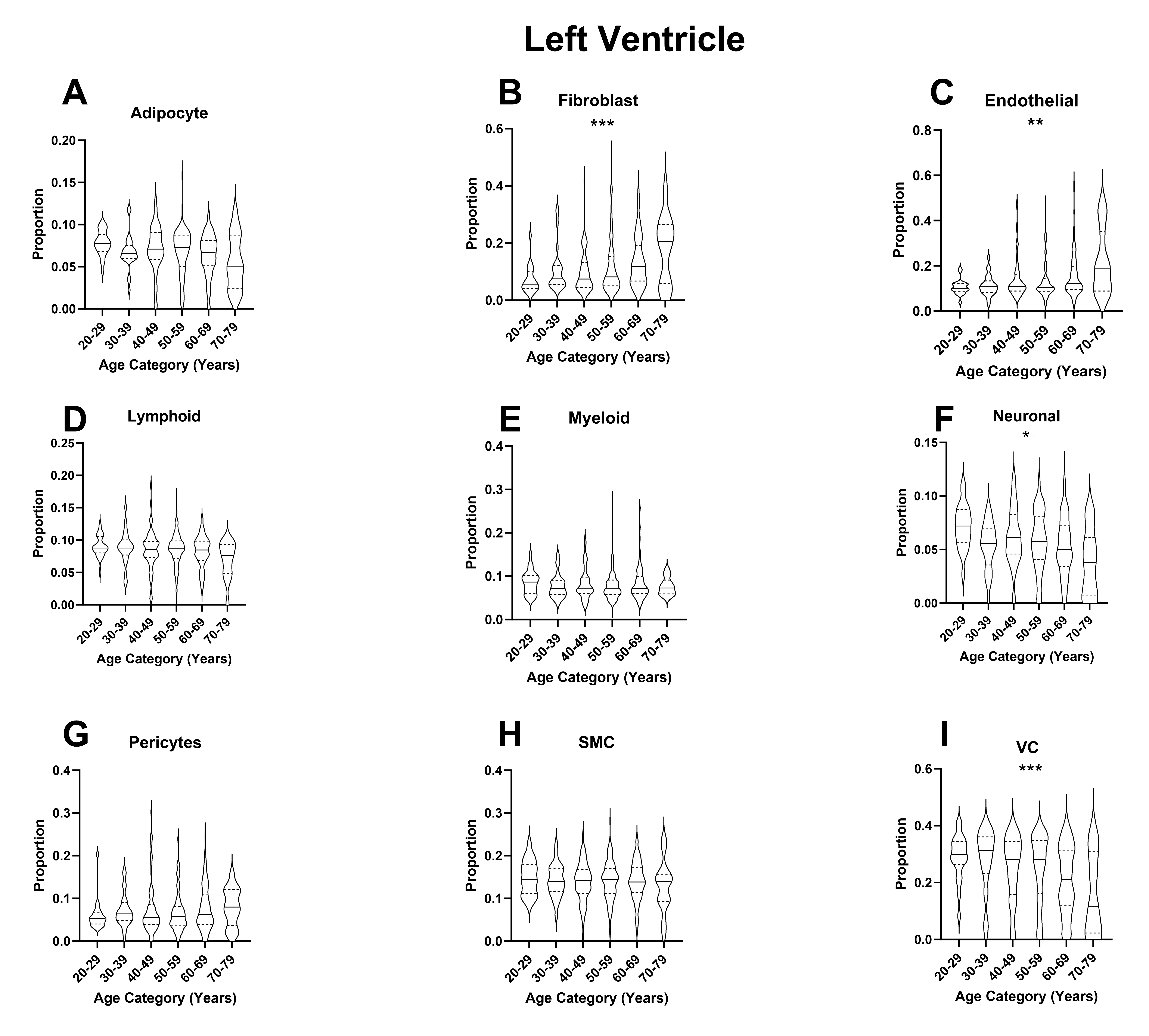


Predicted proportion of cell lineages by CIBERSORTx in LV samples from GTEx per age category. SMC = smooth muscle cells, VC = ventricular cardiomyocytes. * indicate level of statistical significance of difference between groups as determined by Kruskal Wallis test. ** indicates FDR ≤ 0.01, *** indicates FDR ≤0.001.

**Supplementary Figure 9: Age-associated differences in right atrial cell lineage composition in GTEx cohort using CIBERSORTx**


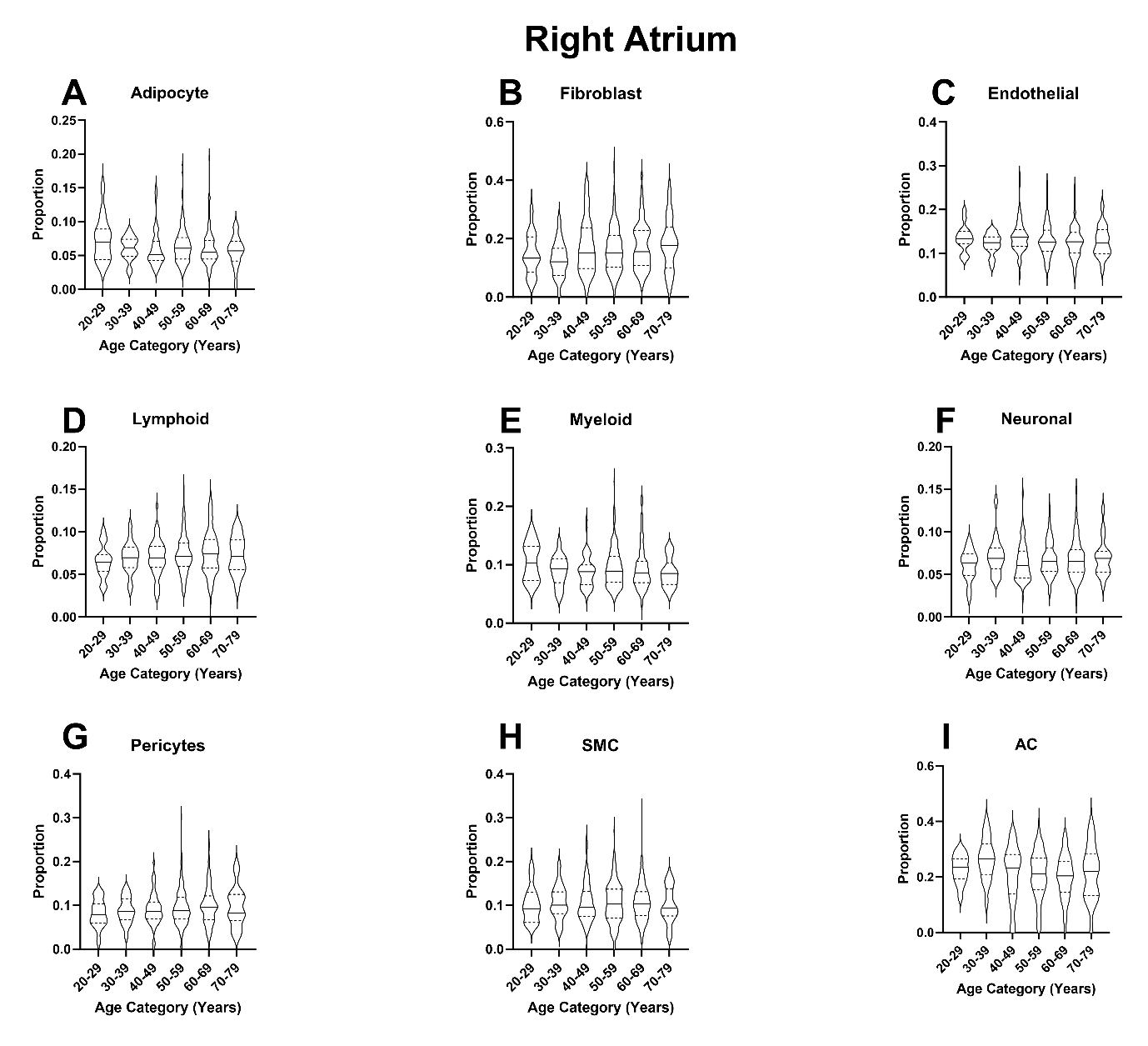


Predicted proportion of cell lineages by CIBERSORTx in RA samples from GTEx per age category. SMC = smooth muscle cells, AC = atrial cardiomyoctyes. * indicates level of statistical significance of difference between groups as determined by Kruskal Wallis test. ** indicates FDR ≤ 0.01, *** indicates FDR ≤0.001.

**Supplementary Figure 10: Age-associated differences in left ventricular cell lineage composition in GTEx cohort using BayesPrism**


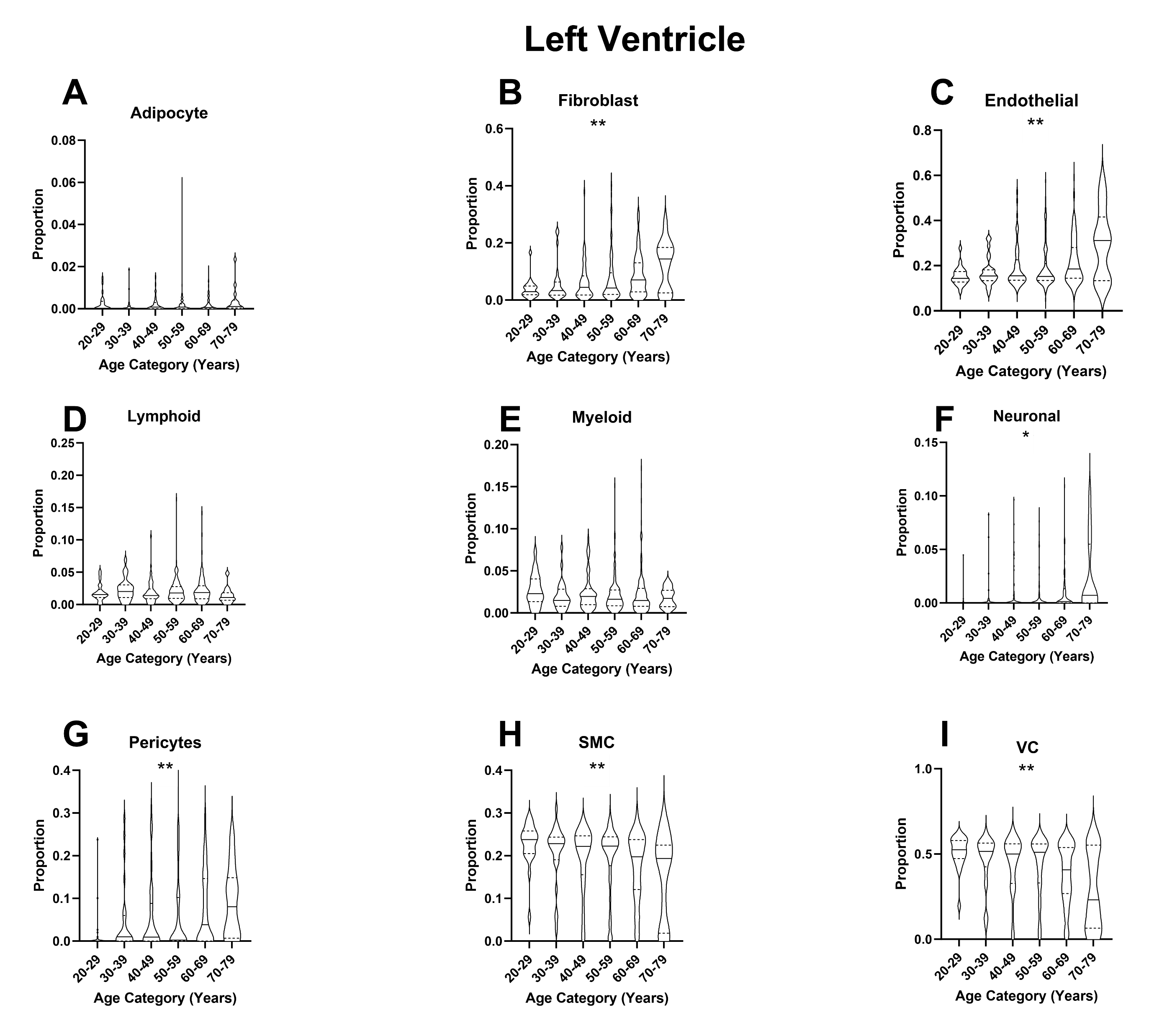


Predicted proportion of cell lineages by BayesPrism in LV samples from GTEx per age category. SMC = smooth muscle cells, VC = ventricular cardiomyocytes. * indicate level of statistical significance of difference between groups as determined by Kruskal Wallis test. ** indicates FDR ≤ 0.01, *** indicates FDR ≤0.001.

**Supplementary Figure 11: Age-associated differences in right atrial cell lineage composition in GTEx cohort using BayesPrism**


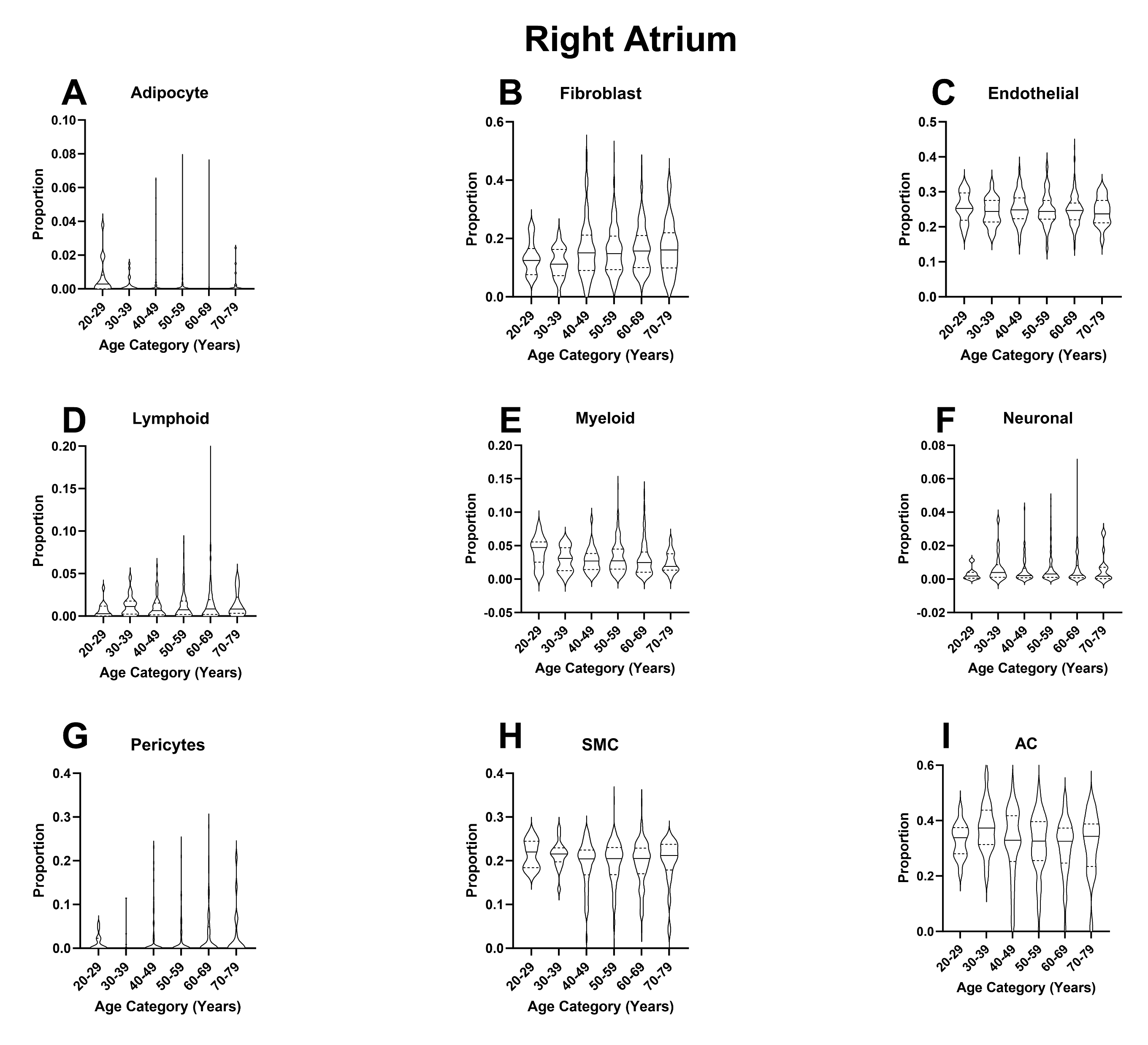


Predicted proportion of cell lineages by BayesPrism in RA samples from GTEx per age category. SMC = smooth muscle cells, AC = atrial cardiomyoctyes. * indicates level of statistical significance of difference between groups as determined by Kruskal Wallis test. ** indicates FDR ≤ 0.01, *** indicates FDR ≤0.001.

**Supplementary Figure 12: Comparison of cell lineage predictions produced by CIBERSORTx and BayesPrism using GTEx left ventricular data**


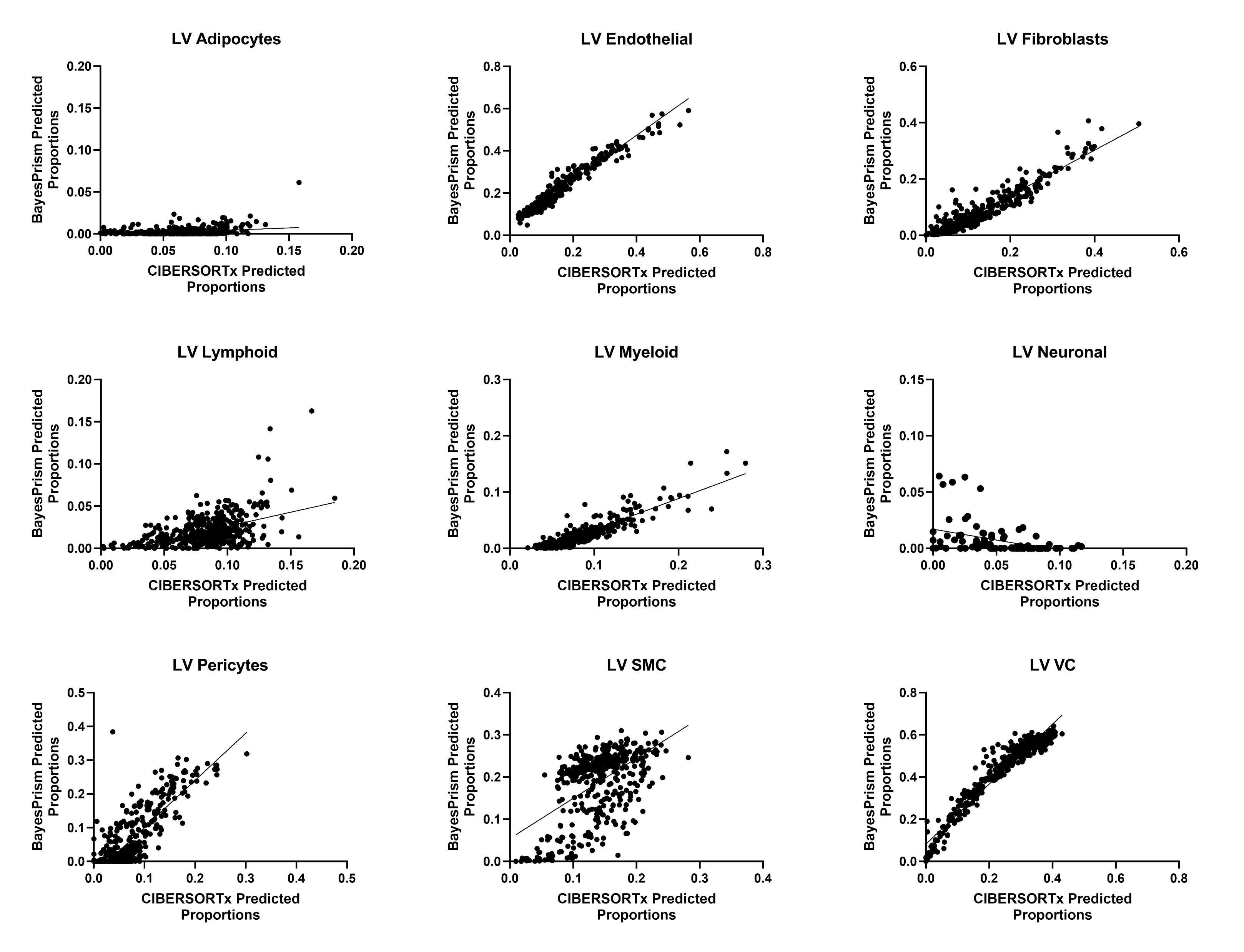


CIBERSORTx versus BayesPrism cell lineage predictions in left ventricular (LV) data from GTEx (n=429). Co-efficients of determination (R^2^) for each lineage are: Adipocyte = 0.10; Fibroblast = 0.90; Endothelial = 0.94; Lymphoid = 0.23; Myeloid = 0.81; Neuronal = 0.17; Pericyte = 0.67; smooth muscle cell (SMC) = 0.55; ventricular cardiomyocyte (VC) = 0.94.

**Supplementary Figure 13: Comparison of cell lineage predictions produced by CIBERSORTx and BayesPrism using GTEx right atrial data**


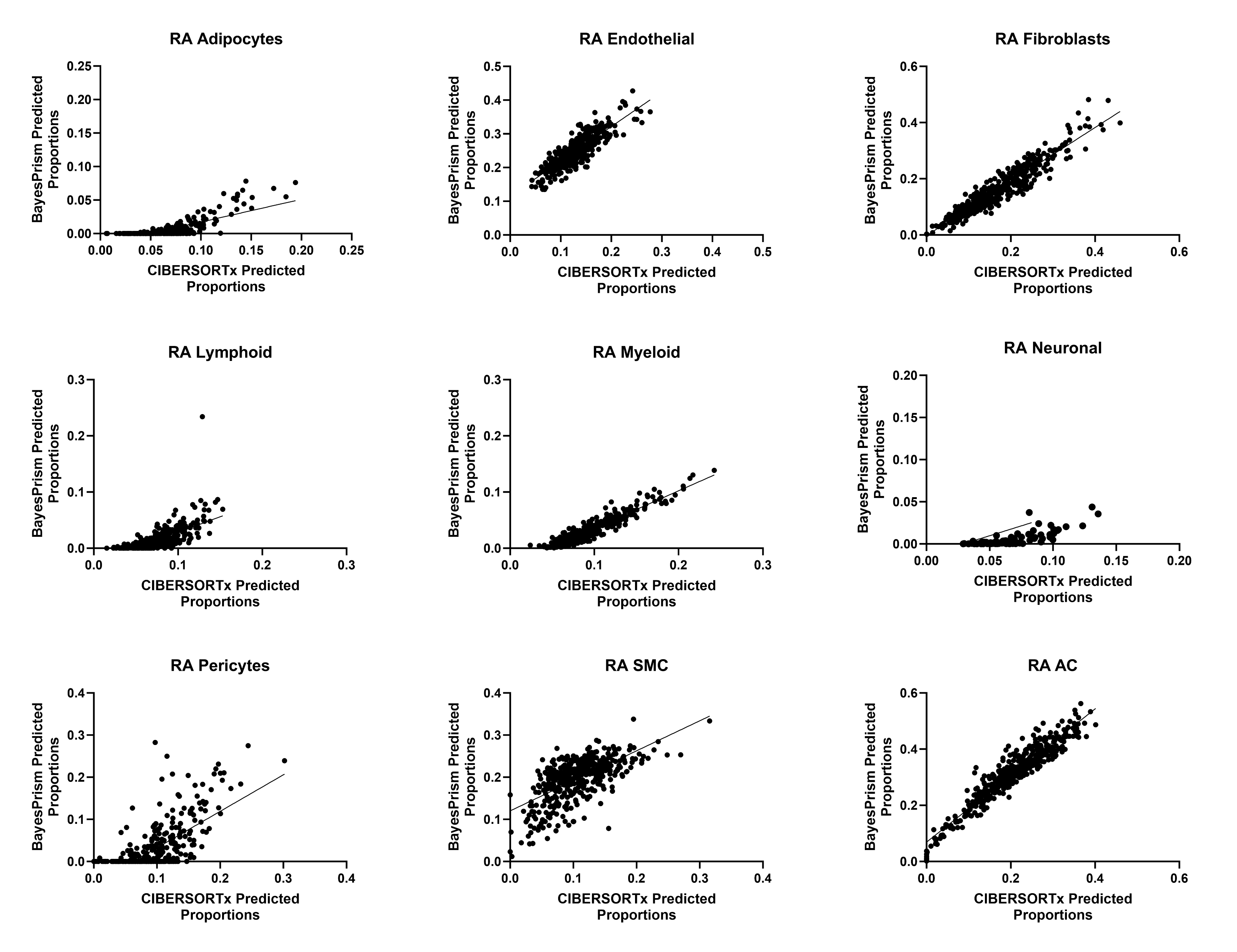


CIBERSORTx versus BayesPrism cell lineage predictions in right atrial (RA) data from GTEx (n=425). Co-efficients of determination (R^2^) for each lineage are: Adipocyte = 0.60; Fibroblast = 0.89; Endothelial = 0.72; Lymphoid = 0.48; Myeloid = 0.89; Neuronal = 0.58; Pericyte = 0.45; smooth muscle cell (SMC) = 0.40; atrial cardiomyocyte (AC) = 0.91.

**Supplementary Figure 14: Sex-associated differences in left ventricular cell lineage composition in GTEx cohort using CIBERSORTx**


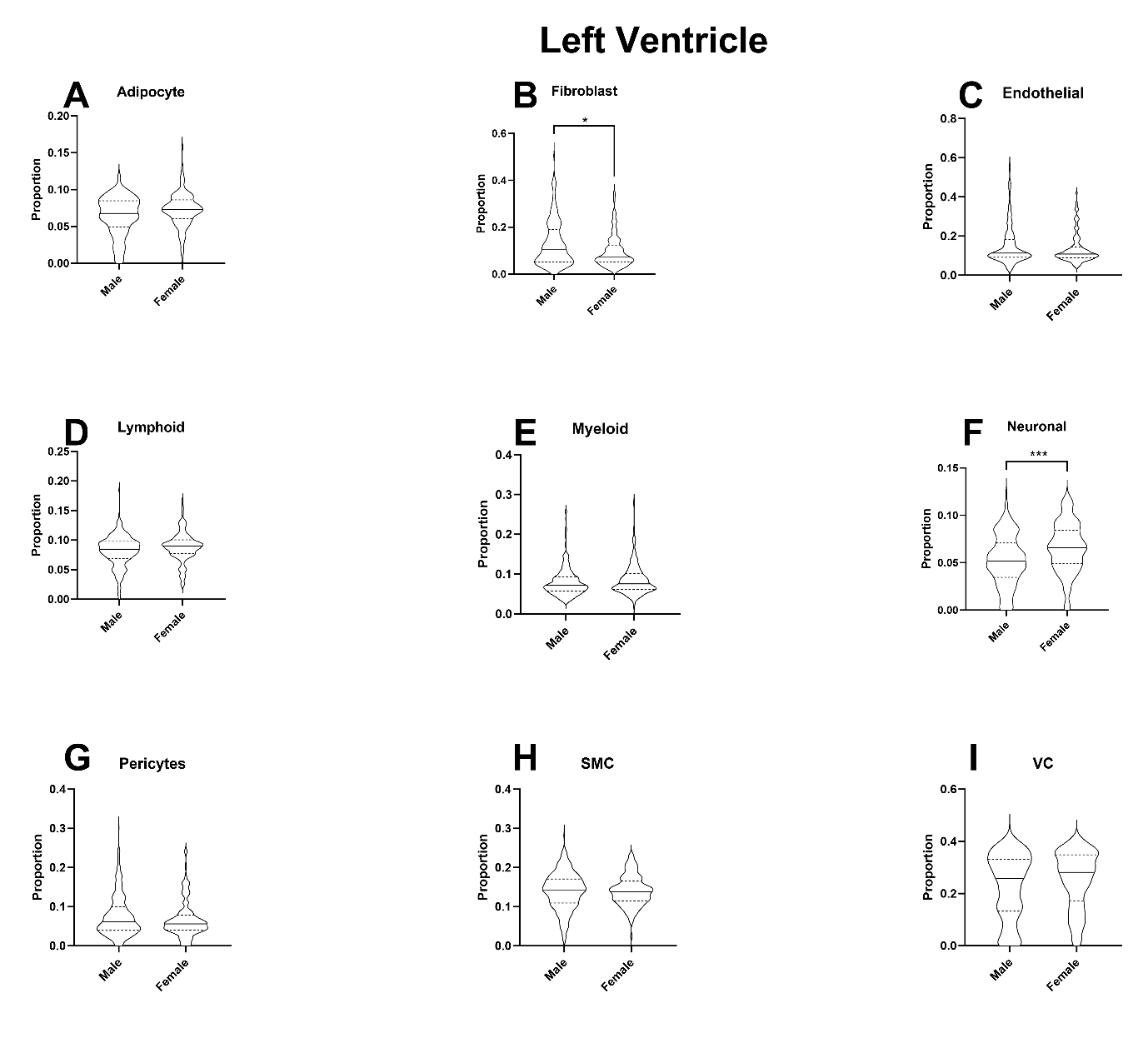


Predicted proportion of cell lineages by CIBERSORTx in LV samples from GTEx in males vs. females. SMC = Smooth muscle cells, VC = ventricular cardiomyocytes * indicate level of statistical significance of difference between groups as determined by Wilcoxon test. * indicates FDR ≤ 0.05, ** indicates FDR ≤ 0.01, *** indicates FDR ≤ 0.001.

**Supplementary Figure 15: Sex-associated differences in left ventricular cell lineage composition in GTEx cohort using BayesPrism**

**
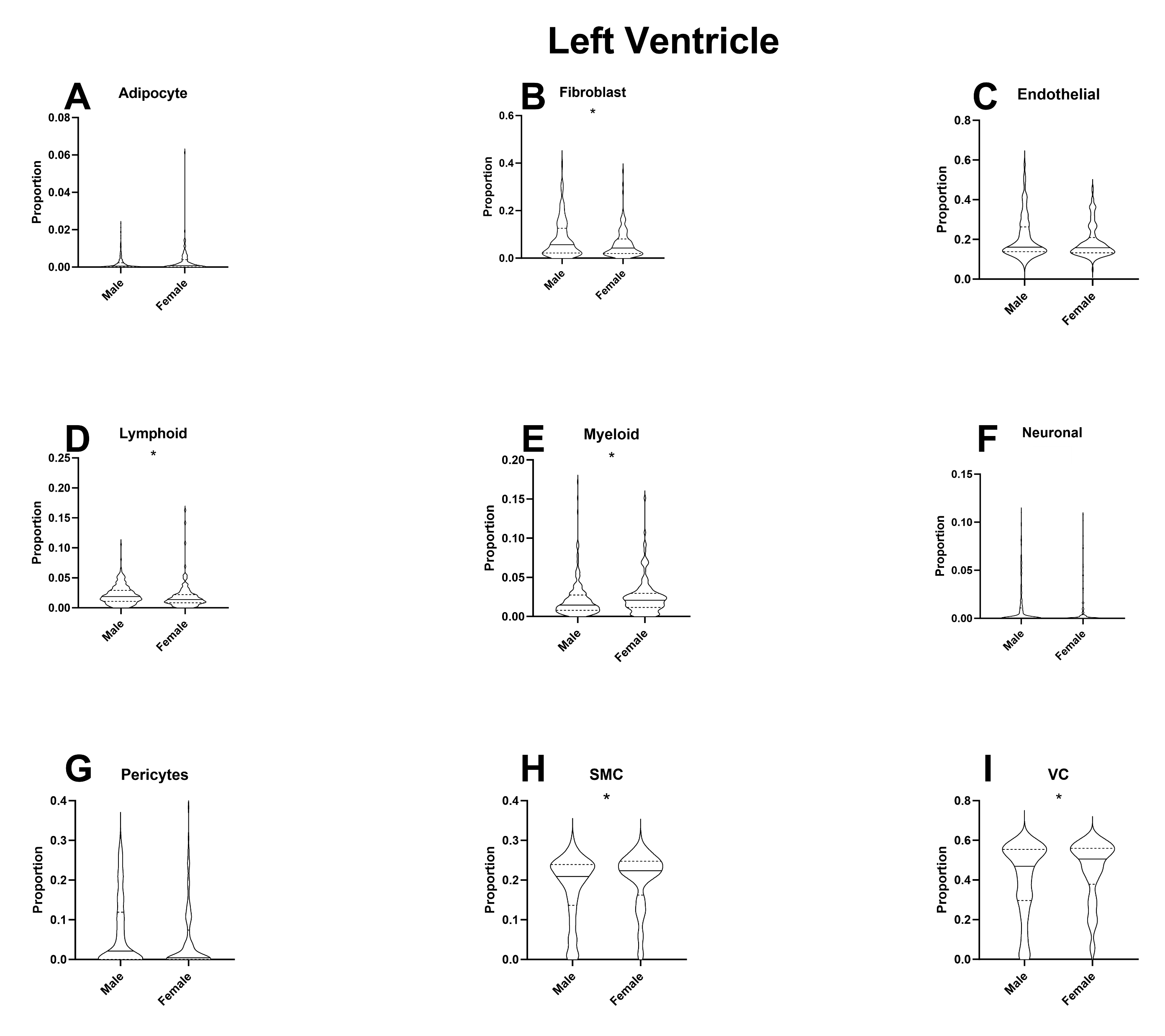
**

Predicted proportion of cell lineages by BayesPrism in LV samples from GTEx in males vs. females. SMC = Smooth muscle cells, VC = ventricular cardiomyocytes * indicate level of statistical significance of difference between groups as determined by Wilcoxon test. * indicates FDR ≤ 0.05, ** indicates FDR ≤ 0.01, *** indicates FDR ≤ 0.001.

**Supplementary Figure 16: Sex-associated differences in right atrial cell lineage composition in GTEx cohort using CIBERSORTx**


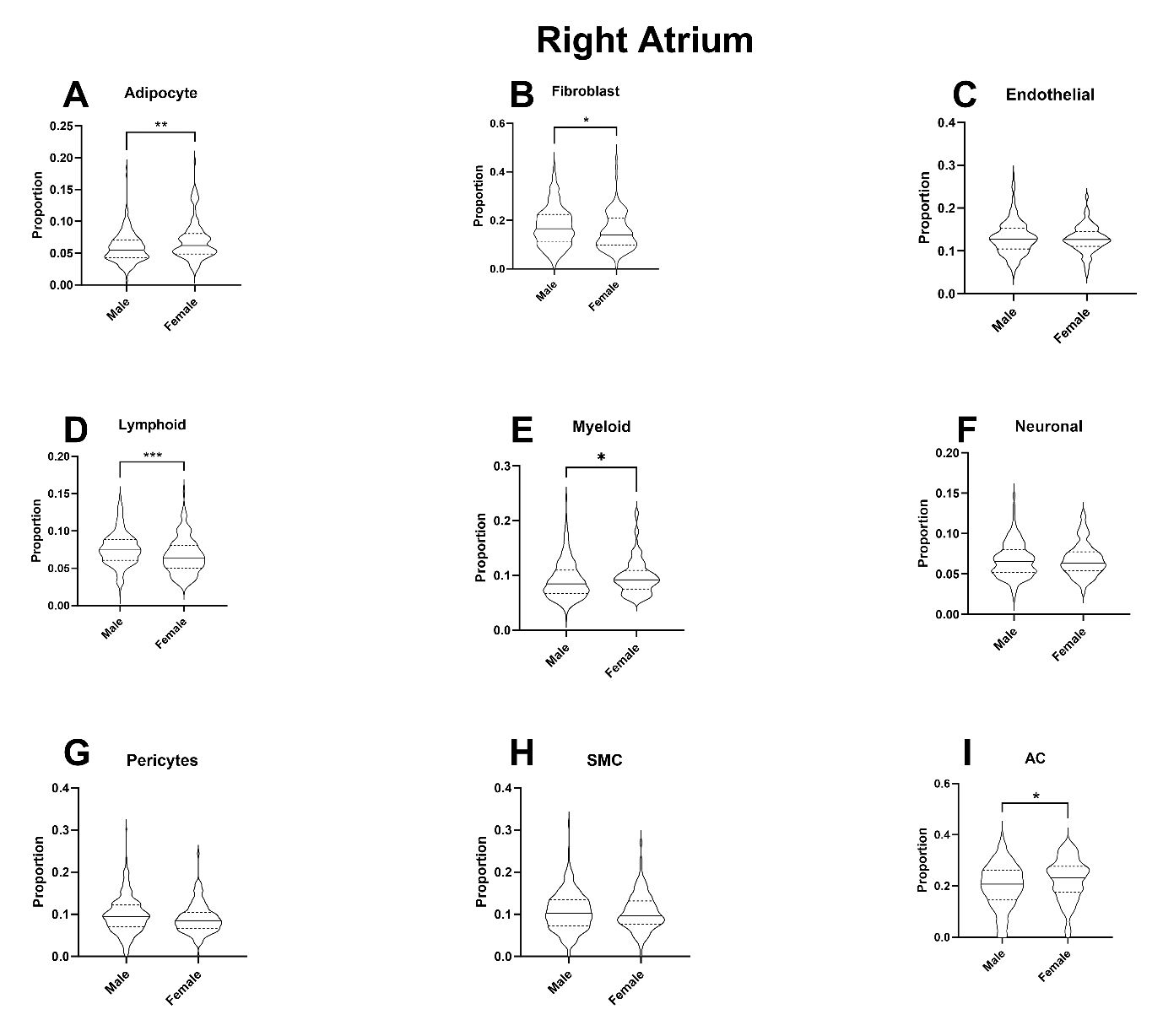


Predicted proportion of cell lineages by CIBERSORTx in RA samples from GTEx in males vs. females. SMC = Smooth muscle cells, AC = atrial cardiomyocytes * indicates level of statistical significance of difference between groups as determined by Wilcoxon test. * indicates FDR ≤ 0.05, ** indicates FDR ≤ 0.01, *** indicates FDR ≤0.001

**Supplementary Figure 17: Sex-associated differences in right atrial cell lineage composition in GTEx cohort using BayesPrism**


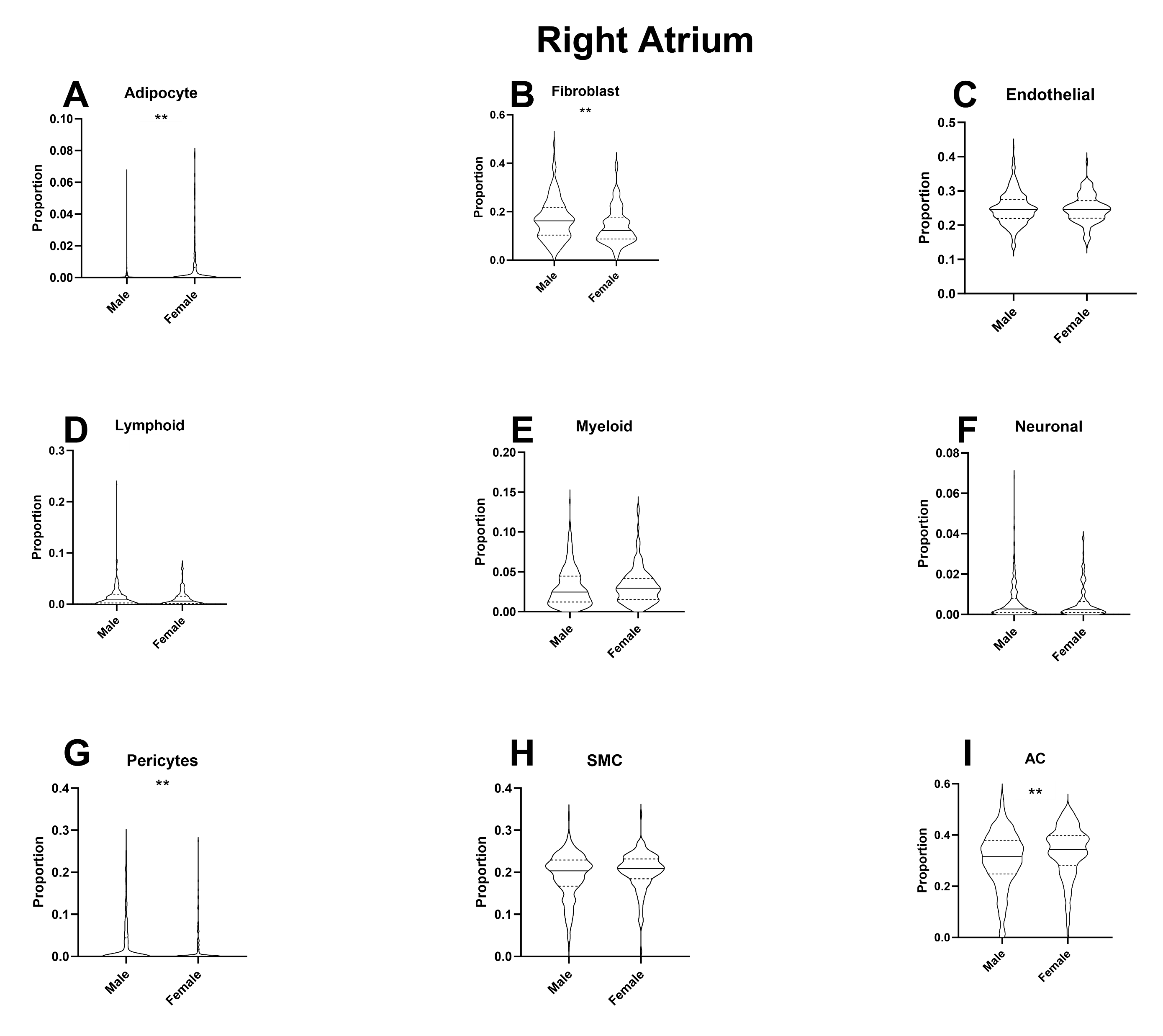


Predicted proportion of cell lineages by BayesPrism in RA samples from GTEx in males vs. females. SMC = Smooth muscle cells, AC = atrial cardiomyocytes * indicates level of statistical significance of difference between groups as determined by Wilcoxon test. * indicates FDR ≤ 0.05, ** indicates FDR ≤ 0.01, *** indicates FDR ≤0.001

**Supplementary Figure 18: Comparison of cell lineage predictions produced by BayesPrism trained using 200 cells per lineage versus all Heart Cell Atlas data in analysis of GTEx right atrial data**


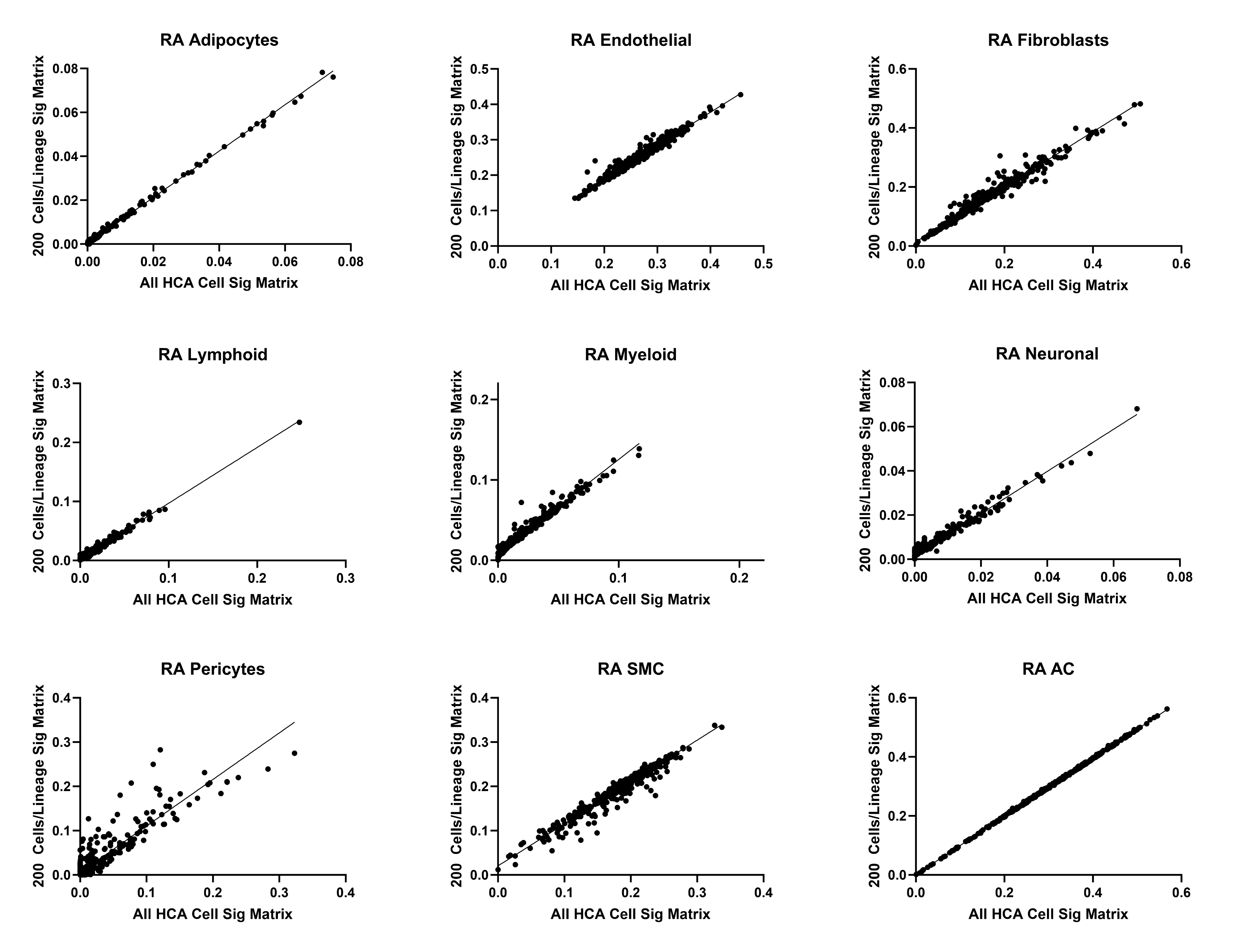


Estimates of cell lineage predictions in right atrial (RA) data from GTEx (n=425) using 200 cells per lineage versus all Heart Cell Atlas right atrial data (except mesothelial cells) to train BayesPrism. Co-efficients of determination (R^2^) for each lineage are: Adipocyte = 0.998; Fibroblast = 0.969; Endothelial = 0.959; Lymphoid = 0.979; Myeloid = 0.966; Neuronal = 0.970; Pericyte = 0.838; smooth muscle cell (SMC) = 0.947; atrial cardiomyocyte (AC) = 0.999.
