# Supplementary material for: Application of CIBERSORTx and BayesPrism to deconvolution of bulk RNA-seq data from human myocardium and skeletal muscle
Source: Heliyon. 2025 Feb 10;11(4):e42499. doi: 10.1016/j.heliyon.2025.e42499 (PMC11872574; doi:10.1016/j.heliyon.2025.e42499)

**CIBERSORTx workflow**

After creating an account at <https://cibersortx.stanford.edu>, the instructions below describe how to use the CIBERSORTx web server to use the source files we provide for deconvolution of any bulk RNA-seq data from human left ventricle, right atrium or skeletal muscle.

Upload Files

- In the add files section press the green Add files… button
- Select the gene expression profile (.GEP) file you want to upload. Supplementary Files 5, 7 and 9 are the source .GEP files for left ventricle, right atrium and skeletal muscle, respectively; select the appropriate one for your tissue of interest. Select the file type as Source GEP and press “Start”
- Repeat process for the “Single Cell Reference Matrix”, using supplementary files 1,2,or 3 for left ventricle, right atrium and skeletal muscle, respectively.
- Upload your bulk RNA-seq files as “Mixture” following appropriate formatting (see CIBERSORTx website for formatting requirements: <https://cibersortx.stanford.edu/tutorial.php>)
- An example image after file selection is provided below:


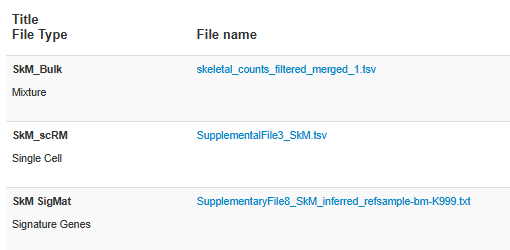


Run CIBERSORTx

- Move to the “Run CIBERSORTx” section of the website
- Under Select Analysis Module: select 2. “Impute Cell Fractions”
- Under Select Analysis Mode: select “Custom”
- Select the previously uploaded Signature Matrix and Mixture files.
- To use the single cell data for batch correction select the tick box for “Enable batch correction” and select “S-mode” and choose the single cell reference matrix file.
- The remaining selections are the user’s choice; however, when using these files we typically select “Disable quantile normalization”, and do not select “Run in absolute mode” and select 500 permutations for significance analysis.
- Press Run
- Output files are found in the “Job Results” area of the website.
- An example image of the preferred settings is provided below:


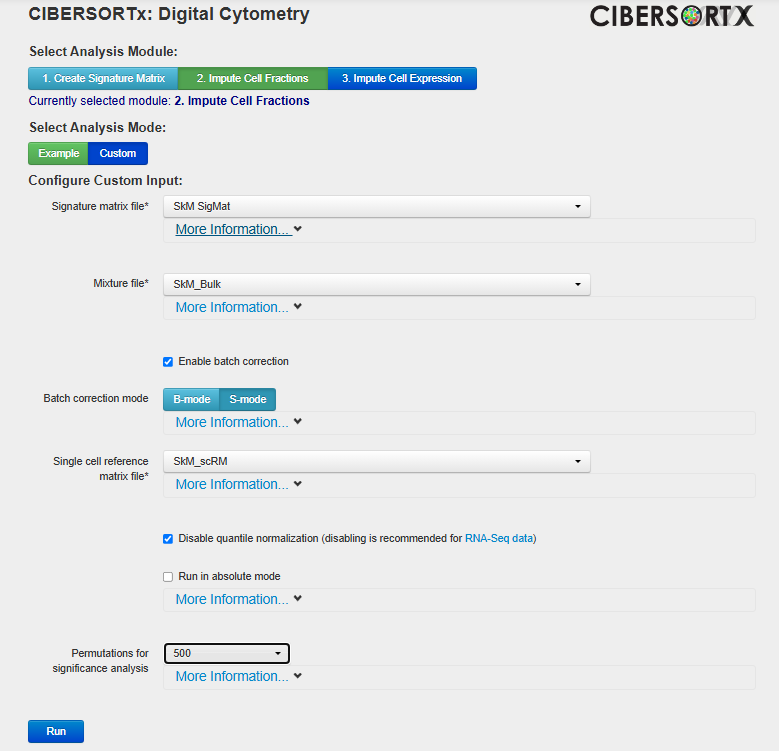

Supplement: Multimedia component 11 [file mmc11.docx]
